# Engineering Flexible Metal‐Phenolic Networks with Guest Responsiveness via Intermolecular Interactions

**DOI:** 10.1002/anie.202302448

**Published:** 2023-03-24

**Authors:** Wanjun Xu, Shuaijun Pan, Benjamin B. Noble, Zhixing Lin, Sukhvir Kaur Bhangu, Chan‐Jin Kim, Jingqu Chen, Yiyuan Han, Irene Yarovsky, Frank Caruso

**Affiliations:** ^1^ Department of Chemical Engineering The University of Melbourne Parkville Victoria 3010 Australia; ^2^ State Key Laboratory of Chemo/Biosensing and Chemometrics and College of Chemistry and Chemical Engineering Hunan University Changsha 410082 China; ^3^ School of Engineering RMIT University Melbourne Victoria 3001 Australia

**Keywords:** Competitive Coordination Assembly, Guest-Responsive Materials, Metal-Organic Frameworks, Microcapsules, Supramolecular Chemistry

## Abstract

Flexible metal‐organic materials are of growing interest owing to their ability to undergo reversible structural transformations under external stimuli. Here, we report flexible metal‐phenolic networks (MPNs) featuring stimuli‐responsive behavior to diverse solute guests. The competitive coordination of metal ions to phenolic ligands of multiple coordination sites and solute guests (e.g., glucose) primarily determines the responsive behavior of the MPNs, as revealed experimentally and computationally. Glucose molecules can be embedded into the dynamic MPNs upon mixing, leading to the reconfiguration of the metal‐organic networks and thus changes in their physicochemical properties for targeting applications. This study expands the library of stimuli‐responsive flexible metal‐organic materials and the understanding of intermolecular interactions between metal‐organic materials and solute guests, which is essential for the rational design of responsive materials for various applications.

## Graphical abstract

Competitive coordination of metal ions to phenolic ligands with multiple coordination sites and solute guests results in flexible metal‐phenolic networks featuring guest‐responsive behavior. This leads to a significant transformation in their coordination networks and hence properties, for example, controllable cargo release, and expands the rational design of flexible metal‐organic materials.

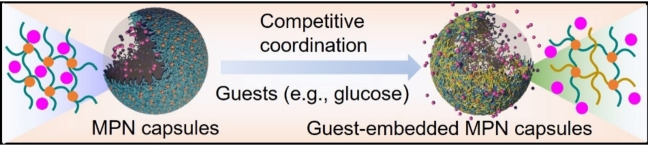

## Introduction

Stimuli‐responsive supramolecular materials play a pivotal role in various fundamental studies and applications across diverse disciplines, including chemistry, biology, and materials science.^[1]^ In particular, supramolecular metal‐organic networks have attracted widespread interest owing to their tunable coordination frameworks and hybrid physiochemical properties originating from metal ions and organic ligands.^[2]^ Efforts have been made to endow rigid metal‐organic materials with stimuli‐responsive behavior via specific ligands,^[3]^ surface modification,^[4]^ and cargo loading.^[5]^ More recently, flexible metal‐organic networks with intrinsic molecular mobility have attracted increasing attention because they can undergo reversible structural transformations under external stimuli, leading to changes in physiochemical properties and enhanced performance for various applications.^[6]^ Despite recent advances, there remains a need to expand the library of stimuli‐responsive flexible metal‐organic network materials and to reveal the molecular mechanisms that underpin their responsive behavior.

Metal‐phenolic networks (MPNs) are supramolecular coordination networks composed of metal ions (e.g., Fe^II^, Al^III^) and phenolic ligands (e.g., tannic acid (TA)). The abundant and asymmetric phenolic ligands enable the formation of flexible metal‐organic network materials with modular properties (e.g., thermal stability, pH‐responsive disassembly, and biocompatibility), which are promising for bionanotechnology and nanoarchitectonics.^[7]^ The supramolecular dynamics of MPNs has been extensively exploited for pH responsiveness and recently for thermal responsiveness.^[8]^ However, the guest‐based responsiveness of MPNs is yet to be explored, and a comprehensive understanding of their intermolecular interactions would promote the rational design of flexible metal‐organic materials with advanced functionalities for diverse applications.

**Figure 1:**
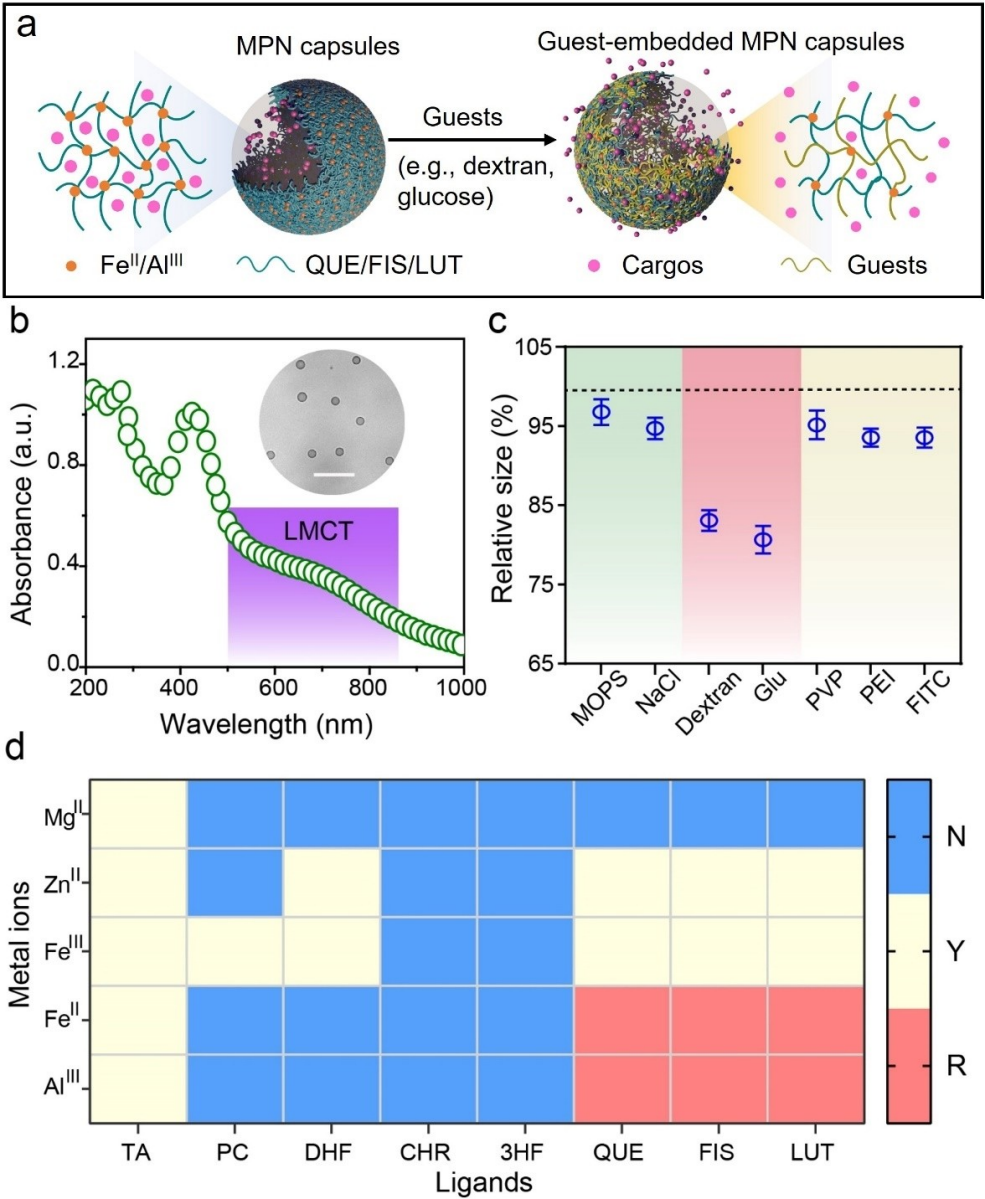
a) Schematic of structural re‐conformation of MPN capsules when subjected to external guest stimuli. b) UV–vis spectrum of Fe^II^‐QUE MPN capsules prepared at pH 4. The violet region highlights the LMCT band. Inset shows the corresponding DIC microscopy image of the monodisperse MPN capsules; scale bar is 5 μm. c) Relative size of Fe^II^‐QUE MPN capsules when dispersed in different solutions. The dashed line represents the template size (i.e., 3.2 μm normalized to 100 %), and the size [%] of the capsules is measured relative to the template size (100 %). MOPS, 3‐(*N*‐morpholino)propanesulfonic acid; Glu, glucose; PVP, polyvinylpyrrolidone, *M*_w_ 10 000; PEI, polyethyleneimine, *M*_w_ 8000; FITC, fluorescein isothiocyanate. Data are shown as the mean±standard deviation (*n*=20). d) Heat map showing the formation of MPN capsules from different metal ions and phenolic ligands and their corresponding responsiveness to 100 mM Glu solution. N, no capsule formed; Y, capsules formed; and R, capsules formed with Glu‐responsive behavior.

Herein, we report the guest‐responsive behaviors of flexible MPNs and reveal their intermolecular competitive coordination‐based responsive mechanism experimentally and computationally. The responsive behavior could be controlled by different building blocks (i.e., metal ions and phenolic ligands) and stimuli guests (e.g., salts, saccharides) (Figure S1). In particular, MPN networks composed of Fe^II^ and quercetin (QUE), i.e., Fe^II^‐QUE MPNs, with significant guest‐responsive behavior, and glucose (Glu), a common and key stimulus in biomedicine, were selected as models to systematically investigate the molecular mechanism underlying their responsive behavior (Figure 1a). Both the simulation and experimental studies showed that the binding affinity between Glu and Fe^II^ was comparable to that between QUE and Fe^II^ (i.e., MPN building blocks), thereby facilitating incorporation of Glu in MPNs and changes in physiochemical properties. The cargo‐loaded (e.g., insulin, INS) MPNs were therefore responsive to solute guests and released cargo. The present study expands the realm of responsive MPNs and provides a mechanistic understanding (i.e., intermolecular competitive coordination) for the rational design of flexible metal‐organic networks for various applications in chemical, biomedical, and material sciences.

## Results and Discussion

The responsiveness of MPNs to different external guest stimuli was first investigated, and MPN capsules were selected as the model owing to their ability to undergo morphological transformation under stimuli.^[8d]^ The MPN capsules were obtained using polystyrene (PS) particles as sacrificial templates. Differential interference contrast (DIC) analysis along with the presence of a broad ligand‐to‐metal charge‐transfer (LMCT) band in the UV–vis spectrum confirmed the formation of spherical Fe^II^‐QUE MPN capsules (Figure 1b). These MPN capsules were then exposed to different external guest stimuli with their pristine pHs (i.e., salts, saccharides, and polymer solutions), except for specific buffers. The Fe^II^‐QUE MPN capsules showed significant size shrinkage toward saccharides including dextran and Glu. For example, the MPN capsules shrunk to 2.4±0.1 μm (25 % shrinkage relative to PS templates) when dispersed in 100 mM Glu solution (Figure 1c). The degree of capsule shrinkage was also dependent on the molecular weight (*M*_w_) of dextran (*M*_w_=3–250 kDa) and concentration of Glu (5–100 mM) (Figure S2). Furthermore, an increase in the Fe^II^‐to‐QUE ratio from 1 : 5 to 2 : 1 significantly enhanced the responsiveness of the capsules (e.g., from 12 % to 25 % shrinkage), whereas no significant changes in size occurred upon further increasing the molar ratio from 2 : 1 to 8 : 1 (Figure S3). Thus, a Fe^II^‐to‐QUE molar ratio of 2 : 1 was established as the optimal ratio for constructing MPN capsules for the subsequent studies.

Phenolic ligands with single or multimodal coordination sites (Figure S4), along with a wide range of metal ions, were then selected to fabricate MPN capsules to reveal the underlying structural requirements of Glu‐responsive behavior. The formation of MPN coatings on PS particles was observed by a shift in ζ‐potential and changes in the suspension color upon coating (Figure S5). Specifically, ligands containing a catechol group (i.e., pyrocatechol (PC) and 3′,4′‐dihydroxyflavone (DHF)) or catechol and galloyl groups (i.e., TA) (single coordination site) coordinated with selected or all examined metal ions generated free‐standing capsules after template removal with none exhibiting size responsiveness to Glu (Figure 1d). The use of ligands containing only hydroxy ketone groups (i.e., chrysin (CHR) and 3‐hydroxyflavone (3HF)) did not result in capsule formation at pH 4 due to insufficient cross‐linking density. In contrast, the use of ligands containing both catechol and hydroxy ketone groups (i.e., QUE, fisetin (FIS), and luteolin (LUT)) (multimodal coordination sites) resulted in intact capsules with most metal ions except Mg^II^, with two metal ion‐based (Al^III^ and Fe^II^) systems showing guest‐responsive behaviors (Figure S6).^[9]^ Collectively, these results suggest that ligands with multimodal coordination sites and metal ions with appropriate binding affinity are essential for fabricating Glu‐responsive MPNs.

**Figure 2:**
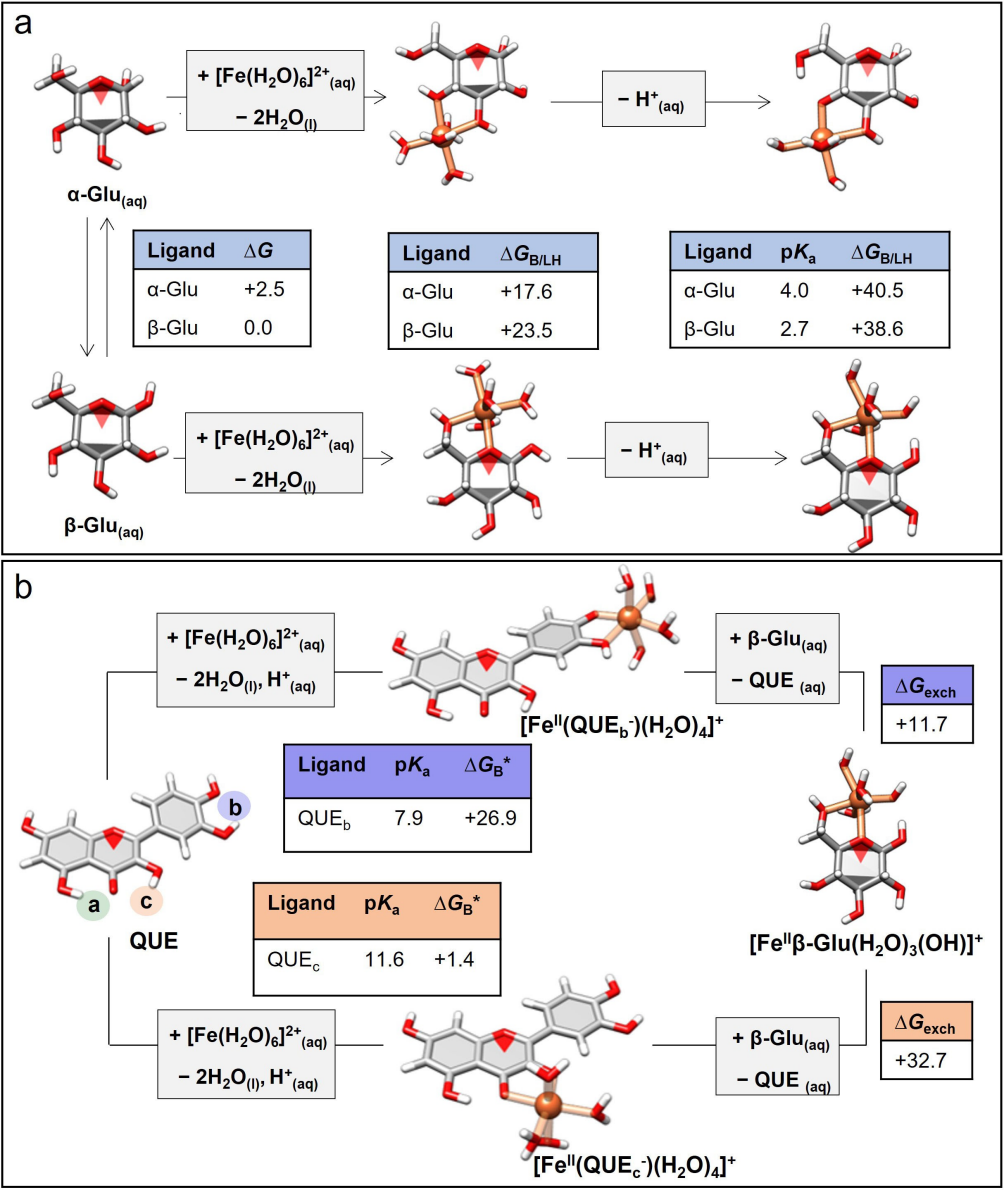
a) Optimized solution‐phase structures and predicted energetics for the chelation of Fe^II^ to α‐Glu or β‐Glu. The binding energy values (kJ mol^−1^) were calculated at 25 °C. All energy values given are relative to β‐Glu. b) Predicted energetics for the exchange of Fe^II^ from the catechol site (site b) and maltol site (site c) of QUE to β‐Glu. The binding and exchange energy values (kJ mol^−1^) were calculated at pH 4 at 25 °C. Δ*G*_B/LH_ defines the strength of binding of a protonated ligand (LH). Δ*G*_B_* encompasses both binding and deprotonation processes. Δ*G*_exch_ describes the energetics of QUE/Glu ligand exchange, that is, replacement of QUE with Glu. aq, aqueous; exch, exchange.

Subsequently, the Fe^II^‐QUE MPNs with multimodal coordination modes and guest‐responsive behavior were chosen as the model to simulate their molecular interaction with guest molecules. The Gibbs free binding energies (Δ*G*) and p*K*_a_ values between Fe^II^ to Glu and Fe^II^ to QUE were predicted from ab initio calculations using ORCA 5.0.1.^[10]^ Specifically, Δ*G*s of Fe^II^ binding to various sites of α‐Glu and β‐Glu with different deprotonated states (i.e., fully, single, or doubly deprotonated) were first systematically calculated (Figures S7–S9), thus identifying the most energy favorable Fe^II^−Glu complexes (Figure 2a).^[11]^ The binding energy of Fe^II^ to β‐Glu was predicted to be *G*_B_*=38.6 kJ mol^−1^, which is not high but comparable to that of Fe^II^ to QUE (particularly at the catechol sites, *G*_B_*=26.9 kJ mol^−1^) at pH 4 (Figure S10).^[12]^ The predicted energy exchange for Fe^II^ transfer from the catechol site of QUE to β‐Glu was +11.7 kJ mol^−1^ (Figure S10b), which equals the ratio of Fe^II^‐QUE and Fe^II^‐Glu complexes of 114 : 1 at equilibrium under equimolar conditions (Figure 2b). The concentration of Glu is significantly higher than the effective concentration of QUE (>200 : 1), which would nearly completely counteract this underlying energetic preference for QUE complexation, leading to the exchange of QUE and Glu and the re‐conformation of metal‐organic networks.

**Figure 3:**
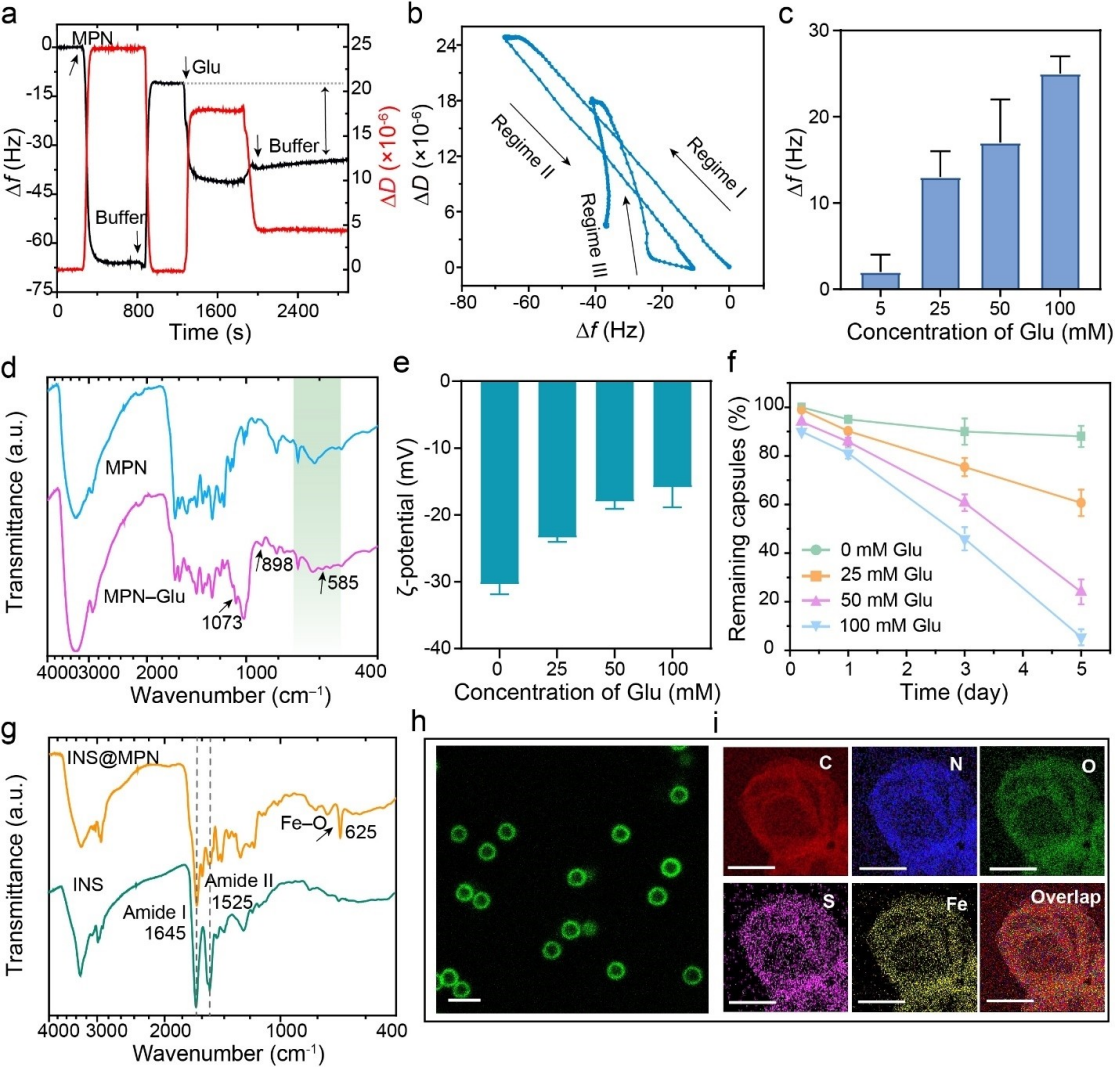
a) Binding kinetics of MPNs with Glu, as monitored by QCM. The double arrow‐dashed line indicates the frequency shift upon addition of Glu. b) Δ*D*–Δ*f* plot illustrating the adhesion dynamics between MPN and Glu. Regime I represents the dynamic adsorption of MPN coatings onto the quartz crystal microbalance chip. Regime II represents the detachment of free MPN coatings. Regime III represents the dynamic adsorption of Glu onto MPN films. c) Frequency shifts of MPNs upon adding Glu at varying concentrations. The error bars represent standard deviations (*n*=3). d) FTIR spectra of MPN and MPN‐Glu capsules. e) Changes in the ζ‐potential of MPN capsules upon addition of Glu at different concentrations (0, 25, 50, and 100 mM). Data are shown as the mean±standard deviation (*n*=3). f) Disassembly kinetics of MPN capsules at different Glu concentrations. The error bars represent standard deviations (*n*=3). g) FTIR spectra of free INS and INS@MPN capsules. h) Confocal laser scanning microscopy (CLSM) image of FINS@MPN capsules. Scale bar is 3 μm. i) EDX elemental mapping of a single INS@MPN capsule. Scale bars are 1 μm.

This competitive coordination and binding kinetics between MPNs and Glu were then monitored in real time by recording the frequency shift (Δ*f*) upon sequential adsorption of related components via quartz crystal microgravimetry (QCM). Specifically, Glu bound preferentially to Fe^II^ than to QUE, as indicated from the relatively higher Δ*f* measured for the binding of Glu to Fe^II^ as opposed to Δ*f*≈0 Hz measured for the binding affinity of Glu to QUE. The binding affinity of Fe^II^ to Glu was slightly higher than that of Fe^II^ to QUE under the preparation conditions employed (Figure S11), as consistent with the prediction from the simulation studies. Moreover, a significant Δ*f* was observed upon addition of Glu solution to the MPN film (Figure 3a), demonstrating the strong interaction between Glu and the MPN networks. The dynamic adsorption and detachment of MPN films in the presence of Glu solution were further characterized by the ratio of the dissipation shift (Δ*D*) to Δ*f*. The resulting Δ*D*/Δ*f* ratio, corresponding to the induced energy loss per unit coupled mass,^[13]^ for regime III (Δ*D*/Δ*f*=−0.91×10^−6^ Hz^−1^) was much higher than that for regime I (Δ*D*/Δ*f*=−0.39×10^−6^ Hz^−1^) (Figure 3b), indicating that the obtained film with Glu embedded within the MPNs is more dissipative and flexible than the pure MPN film. Specifically, Δ*f* was dependent on the concentration of Glu (Figure 3c), in agreement with the responsiveness of the MPN capsules. In contrast, no significant changes in Δ*f* or Δ*D* were observed when introducing NaCl (nonresponsive guest) into the system (Figure S12). The incorporation of Glu within the MPN networks was further characterized by Fourier transform infrared (FTIR) spectroscopy and ζ‐potential measurements. The presence of C−O and C−C stretching vibrations at 1073 and 898 cm^−1^ indicated the existence of Glu on the formed capsules,^[14]^ and the peak at 585 cm^−1^ was ascribed to the coordination bond formed between the hydroxyl groups of Glu and Fe^II^ (Fe−O band, Figure 3d, Table S1).^[15]^ Specifically, the proportion of maltol‐based (C=O→Fe) and catechol‐based (Ph−O→Fe) coordination were lower in Glu‐embedded MPN than in pristine MPN capsules (Figure S13, Table S2), indicating that QUE was partly replaced by Glu in the reconfigured coordination networks. The ζ‐potential of the MPN capsules gradually shifted from −30 mV to −17 mV with the addition of Glu solution at increasing concentrations from 0 to 100 mM owing to the embedding of relatively positively charged Glu into the MPN networks (Figure 3e).^[16]^ Furthermore, the embedding of Glu inside the MPN influenced the stability of the capsules. Therefore, the degradation rate of the MPN capsules could also be engineered by varying the concentration of Glu (Figure S14). For example, in the presence of 100 mM Glu solution, most MPN capsules disassembled within 5 days, whereas in the presence of 25 mM Glu solution, ≈40 % of the capsules disassembled (Figure 3f). Although Glu was incorporated in the preformed MPNs, the direct assembly of Fe^II^‐Glu complexes on PS particles did not yield free‐standing capsules upon template removal (Figure S15), probably owing to their relatively weaker coordination interaction compared with Fe^II^‐QUE complexes (Figure 2). Responsive materials are promising for various applications, including biosensors and drug delivery, as they can load cargos and then release them in a controllable manner at target sites.^[17]^ INS, an important cargo for glucose‐responsive‐based applications, was embedded within the MPN capsules for further controlled release studies. The formation of INS@MPN films resulted in a shift of the ζ‐potential of the carboxyl‐modified PS (PS‐COOH) particles from negative to positive due to the positive charge of INS at pH 4.0 (Figure S16a).^[18]^ DIC and transmission electron microscopy analyses revealed the formation of intact INS@MPN capsules upon PS‐COOH template removal (Figure S16b, c). The presence of Fe−O stretching vibrations at 625 cm^−1^ and the two characteristic peaks at amide I and amide II indicated the incorporation of INS within the capsules (Figure 3g).^[19]^ The fluorescein isothiocyanate‐labeled insulin (FINS) enabled the visualization of the capsules (Figure 3h), and energy‐dispersive X‐ray (EDX) mapping results revealed the presence of elements C, N, O, S, and Fe (Figure 3i), confirming the formation of FINS@MPN capsules.

**Figure 4:**
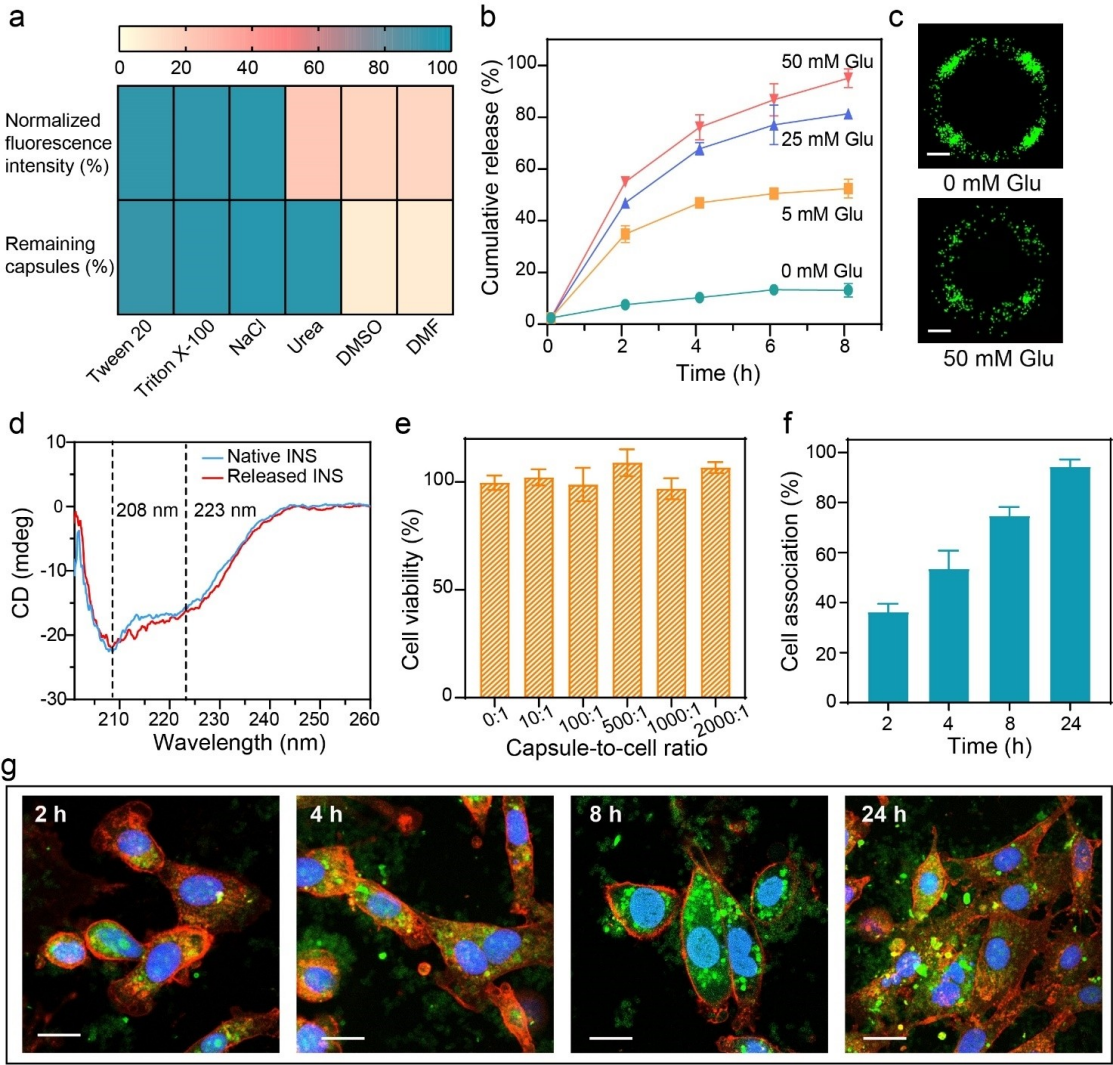
a) Heat map showing the percentage of fluorescence intensity and quantity of MPN capsules after incubation in different media for 1.5 h. Fluorescence intensity was measured by CLSM, and the quantity of capsules was determined by flow cytometry. b) Cumulative release of INS from capsules upon incubation with Glu at different concentrations over time. Data are shown as the mean±standard deviation (*n*=3). c) Stochastic optical reconstruction microscopy images of a single FINS@MPN capsule after incubation with Glu at different concentrations for 8 h; scale bars are 300 nm. d) CD spectra of native INS and INS released from capsules, as triggered by 100 mM Glu. e) Cell viability of 3T3 cells incubated with FINS@MPN capsules at different capsule‐to‐cell ratios. The error bars represent standard deviations (*n*=5). f) Cell association kinetics of FINS@MPN capsules after incubation with 3T3 cells for 2, 4, 8, and 24 h. The error bars represent standard deviations (*n*=3). g) CLSM images of internalization FINS@MPN capsules by 3T3 cells. Green, FINS@MPN capsules; blue, nuclei; and red, cell membrane. Scale bars are 10 μm.

To achieve better controlled release of FINS from capsules upon exposure to external guest stimuli (i.e., Glu), we investigated the fundamental interaction between FINS and MPNs. The FINS@MPN capsules were incubated with hydrophobic competitors (e.g., Tween 20, Triton X‐100), a hydrogen bond competitor (e.g., urea), an ionic interaction competitor (e.g., sodium chloride (NaCl)), and π competitors (e.g., dimethyl sulfoxide (DMSO) and dimethylformamide (DMF)). Note that DMSO and DMF are polar aprotic solvents and can form polar‐π interactions with existing π‐interaction dominant materials, thus acting as π competitors.^[20]^ The FINS@MPN capsules remained stable in 100 mM Tween 20, Triton X‐100, NaCl, and urea, whereas they completely disassembled in DMSO and DMF (Figure 4a, Figure S17). The incubated capsules were then washed and redispersed in water to avoid potential fluorescence quenching. The fluorescence of the capsules remained stable in Tween 20, Triton X‐100, and NaCl but significantly decreased in urea, DMSO, and DMF (Figures S18 and S19). Collectively, these results suggest that FINS is embedded within the MPNs through mainly hydrogen bonding and π interactions.

The release kinetics of cargos from the capsules, as triggered by external Glu stimuli, were then examined. As observed from Figure 4b and Figure S20, the release of FINS from FINS@MPN capsules was dependent on the concentration of Glu, where 92 % of FINS was released at 50 mM Glu whereas only 10 % of FINS was released in the absence of Glu within 8 h. Of particular interest is that the release kinetics of FINS from the MPN capsules can also be tuned by the metal ions owing to their different binding affinity to Glu. For example, in the presence of 5 mM Glu solution, the release rate of FINS decreased from 48 % in Fe^II^‐based MPN capsules (Figure 4b) to 15 % in Al^III^‐based MPN capsules (Figure S21) within 8 h, demonstrating the potential of using responsive MPNs for various applications, which may require specific cargo release profiles.^[21]^ Moreover, a pulsatile release of FINS from the capsules was achieved when the dynamic Glu concentration shifts between hyperglycemic (400 mg dL^−1^) and normoglycemic (100 mg dL^−1^) levels (Figure S22), thereby showing the potential of the present particle platform to effectively regulate physiological Glu levels in real time.

The release kinetics of FINS from the MPN capsules was further visualized by stochastic optical reconstruction microscopy. The number of FINS molecules per capsule was quantified via intensity and automatically counted by Nikon NIS elements software.^[22]^ Around 78 % of FINS were released from the capsules in the presence of 50 mM Glu within 8 h, as consistent with the release profile obtained from fluorescence spectrophotometry (Figure 4c, Figure S23). Note that the appearance of uneven fluorescence signals of the capsule is likely due to only a thin edge of the capsule adjacent to the coverslip being illuminated by a shining laser with a total internal reflection fluorescence angle, while other parts of the capsule are out of the plane and thus not illuminated.^[23]^ Circular dichroism (CD) spectra of the released and native INS both displayed the typical negative bands at 208 and 223 nm, indicating that the secondary conformational structure and function of FINS were not compromised (Figure 4d).^[24]^ The versatility of the current strategy was further demonstrated through the incorporation of another cargo (Cytochrome C) within the MPN capsules—the resulting capsules also exhibited controlled cargo release triggered by Glu solution (Figure S24).

Stimuli‐responsive materials with controllable release are promising for drug delivery. Thus, we further investigated the potential of the guest‐responsive MPNs The INS@MPN capsules showed negligible cytotoxicity at increasing capsule‐to‐cell ratios of up to 2000 : 1 (Figure 4e), which is attributed to the use of natural building blocks. The association of FINS@MPN capsules with 3T3 cells was dependent on the incubation time, and most cells (around 94 %) associated with the capsules after 24 h of incubation (Figure 4f, g and Figure S25). Moreover, more than 60 % of FINS@MPN capsules remained stable in a simulated stomach environment (i.e., gastric fluid containing endopeptidase; pH 2) for 2 h (Figure S26), which is essential for oral drug delivery. In addition, the FINS@MPN capsules exhibited higher transepithelial transport efficiency than free FINS based on the Caco‐2 cell monolayer permeability assay, probably owing to the protection of loaded FINS against a harsh gastrointestinal environment and enhanced FINS intracellular delivery efficiency (Figure S27).^[25]^ Altogether, the cargo‐loaded Glu‐responsive MPN capsules exhibit negligible cytotoxicity, high stability, and transport ability and therefore are potentially promising for various biomedical applications.

## Conclusion

We have developed flexible metal‐organic materials that can respond to various external solute guests, including Glu and dextran. The underlying molecular mechanism of the guest‐responsive behavior of the MPNs was revealed both experimentally and computationally, where ligands with multimodal coordination sites and appropriate binding affinity between ligands and metal ions are dominant factors. Upon exposure to guest stimuli, Glu partially replaced QUE and was embedded into MPNs owing to their comparable intermolecular interactions and therefore led to the re‐conformation of metal‐organic networks and related changes in physiochemical properties. The cargos (e.g., INS) were then incorporated into MPNs and their release was regulated by the external stimuli (e.g., concentration). This work provides insights into the molecular mechanism of stimuli‐responsive behaviors of MPNs and is expected to aid in the rational design of flexible metal‐organic networks for various fields.

## Conflict of interest

The authors declare no conflict of interest.

1

## Supporting information

As a service to our authors and readers, this journal provides supporting information supplied by the authors. Such materials are peer reviewed and may be re‐organized for online delivery, but are not copy‐edited or typeset. Technical support issues arising from supporting information (other than missing files) should be addressed to the authors.

Supporting Information

## Data Availability

The data that support the findings of this study are available from the corresponding author upon reasonable request.
