# Supplementary material for: Engineering Flexible Metal‐Phenolic Networks with Guest Responsiveness via Intermolecular Interactions
Source: Angew Chem Int Ed Engl. 2023 Mar 24;62(18):e202302448. doi: 10.1002/anie.202302448 (PMC10947570; doi:10.1002/anie.202302448)
Supplement: Supplementary file 1 — Supporting Information [file ANIE-62-0-s001.pdf]

Angewandte Chemie
Eine Zeitschrift der Gesellschaft Deutscher Chemiker

Supporting Information

Engineering Flexible Metal-Phenolic Networks with Guest Responsiveness via Intermolecular Interactions

*W. Xu, S. Pan, B. B. Noble, Z. Lin, S. Kaur Bhangu, C.-J. Kim, J. Chen, Y. Han, I. Yarovsky, F. Caruso**

Supporting Information
©Wiley-VCH 2021
69451 Weinheim, Germany

Engineering Flexible Metal–Phenolic Networks with Guest Responsiveness via Intermolecular Interactions

Wanjun Xu, Shuaijun Pan, Benjamin B. Noble, Zhixing Lin, Sukhvir Kaur Bhangu, Chan-Jin Kim, Jingqu Chen, Yiyuan Han, Irene Yarovsky, and Frank Caruso*

Abstract: Flexible metal–organic materials are of growing interest owing to their ability to undergo reversible structural transformations under external stimuli. Here, we report flexible metal–phenolic networks (MPNs) featuring stimuli-responsive behavior to diverse solute guests. The competitive coordination of metal ions to phenolic ligands of multiple coordination sites and solute guests (e.g., glucose) primarily determines the responsive behavior of the MPNs, as revealed experimentally and computationally. Glucose molecules can be embedded into the dynamic MPNs upon mixing, leading to the reconfiguration of the metal–organic networks and thus changes in their physicochemical properties for targeting applications. This study expands the library of stimuli-responsive flexible metal–organic materials and the understanding of intermolecular interactions between metal–organic materials and solute guests, which is essential for the rational design of responsive materials for broader applications.

DOI: 10.1002/anie.2021XXXXX

Section S1. Experimental Procedures

Materials

Quercetin (QUE), luteolin (LUT), fisetin (FIS), 3-hydroxyflavone (3HF), chrysin (CHR), 3′,4′-dihydroxyflavone (DHF), tannic acid (TA), pyrocatechol (PC), iron(II) chloride tetrahydrate ($FeCl_2 \cdot 4H_2O$), iron(III) chloride hexahydrate ($FeCl_3 \cdot 6H_2O$), zinc(II) nitrate hexahydrate ($Zn(NO_3)_2 \cdot 6H_2O$), aluminum(III) chloride hexahydrate ($AlCl_3 \cdot 6H_2O$), magnesium(II) chloride hexahydrate ($MgCl_2 \cdot 6H_2O$), 3-(*N*-morpholino) propanesulfonic acid (MOPS), sodium chloride (NaCl), sodium acetate, fluorescein isothiocyanate (FITC), glucose (Glu), dextran of varying average molecular weights (M_w 3, 6, 20, 70, 110, and 250 kDa), polyvinylpyrrolidone (PVP), polyethyleneimine (PEI), (3-aminopropyl)triethoxysilane (APTES), urea, Triton X-100, Tween 20, Dulbecco's modified Eagle medium (DMEM), Minimum essential medium, Hank's balanced salt solution (HBSS), Dulbecco's phosphate-buffered saline (DPBS), and fetal bovine serum (FBS) were purchased from Sigma-Aldrich (USA). Polystyrene (PS) (D = 3.20 ± 0.13 μm) and carboxyl-modified polystyrene (PS-COOH) (D = 1.86 ± 0.08 μm) particles were purchased from microParticles GmbH. Dimethyl sulfoxide (DMSO), dimethylformamide (DMF), 1,4-dioxane, methanol, and ethanol were purchased from Chem-Supply. Insulin (INS), cytochrome C (CYC), pepsin, trypsin, and chymotrypsin were purchased from Thermo Fisher Scientific (USA). Caco-2 cells and 3T3 cells were purchased from the American Type Culture Collection (ATCC). Transwell inserts were purchased from Corning (USA). Milli-Q water with a resistivity of 18.2 MΩ cm was obtained from a three-stage Millipore Milli-Q plus 185 purification system (Millipore Corporation, USA).

Characterization

Quartz crystal microbalance (QCM) analysis was performed on a Q-Sense E4 quartz crystal microbalance from Biolin Scientific (Västra Frölunda, Sweden), and gold-coated QCM crystals were purchased from ATA Scientific (Taren Point, Australia). Differential interference contrast (DIC) microscopy images of capsules were taken with an inverted Olympus IX71 microscope. Transmission electron microscopy and energy-dispersive X-ray mapping analysis of capsules were performed on an FEI Tecnai TF20 instrument (USA) at an operating voltage of 200 kV. Confocal laser scanning microscopy (CLSM) images were taken with a Nikon A1R+ laser scanning confocal microscope (Nikon Corporation, Japan). ζ-Potential measurements were performed using a Malvern Zetasizer Nano ZS instrument (Malvern Instrument, UK). Fourier transform infrared (FTIR) spectroscopy analysis was conducted on a Tensor–II–FTIR spectrometer. Fluorescence spectra were recorded on a Horiba FluoroLog spectrophotometer at an excitation wavelength of 460 nm. Stochastic optical reconstruction microscopy (STORM) images were acquired on a Nikon N-STORM system equipped with a Nikon 100× 1.4 NA oil immersion objective.

Preparation of Metal–Phenolic Network (MPN) Capsules from Particle Templates

All phenolic ligands and metal ion solutions were prepared freshly for immediate use. The standard protocol used for capsule preparation was as follows: PS particles (30 μL) were washed twice with Milli-Q water (2000 *g*, 1 min) and then suspended in water (365 μL). Then, methanol (75 μL), $FeCl_2 \cdot 4H_2O$ (20 μL, 10 mg mL^{-1} in water), and QUE (30 μL, 5 mg mL^{-1} in methanol) were added successively to the PS dispersion at room temperature (25 °C), followed by brief vortexing and sonication. The pH of the mixed dispersion was adjusted by adding MOPS (500 μL, 100 mM; pH 4) and the suspension was vortexed for 60 s. The mixture was allowed to sit undisturbed for 2 h to achieve sufficient film formation and adherence. Noncoating complexes were removed by centrifugation (2000 *g*, 5 min) and the pellet was washed three times with Milli-Q water. To obtain MPN capsules, 1,4-dioxane (1000 μL) was added to dilute the suspension and for subsequent particle incubation for at least 1 h. Then, the pellets were washed four times with 1,4-dioxane (2000 *g*, 5 min), and the resulting capsules were resuspended in water (200 μL) for characterization.

For the preparation of MPN coatings using other building blocks, the concentration of the metal ions was 10 mg mL^{-1}, and that of the ligands was 2.5 mg mL^{-1} (3HF, CHR, or DHF), 5 mg mL^{-1} (FIS or LUT), 5 mg mL^{-1} (PC), or 10 mg mL^{-1} (TA). The metal ion-to-ligand molar ratio was fixed at 2:1, and the same fabrication process was applied.

Dynamic Size Measurements

For the dynamic size measurements, the MPN-coated PS suspension (2 μL) was added to an APTES-modified microscopy glass substrate and then the substrate was kept still for 1 min. Then, 1,4-dioxane (200 μL) was added in situ to remove the PS templates, and excess 1,4-dioxane was aspirated. Subsequently, MOPS (200 μL, 100 mM; pH 7), NaCl, dextran (M_w = 3 kDa), Glu, PVP, PEI, or FITC solution was added to the capsule area on the glass substrate and incubated for 10 min. To measure the MPN capsule size in the above solutions, the diameter of the capsules (n = 20) was measured by DIC microscopy.

Quantum Mechanical (QM) Methodology

The computational methodology and software packages used to calculate Gibbs free energies are the same as those employed in the previous work.[1] All density functional theory (DFT) and wavefunction calculations were performed in ORCA 5.0.1.[2] Geometry optimizations were performed using the B97M-V functional[3] and def2-TZVP basis set,[4] using the resolution of the identity (RIJ) approximation and a def2/J auxiliary basis set.[5] These optimizations were performed directly in a conductor-like polarizable continuum model (CPCM) solvent field,[6] with water chosen as the solvent. Improved energies were calculated with DLPNO-CCSD(T) and the cc-pVTZ basis set,[7] using the RIJCOSX approximation and the cc-pVTZ/C and def2/J auxiliary basis sets.[5,8] These DLPNO-CCSD(T) single point calculations were also performed in a CPCM solvent field. All DFT and DLPNO-CCSD(T) calculations were performed using "Tight" Self-Consistent Field (SCF) cutoffs.

Having optimized the structures, (numerical) frequencies were calculated in CPCM solution to confirm that structures were true minima, possessing no imaginary frequencies. Solution-phase Gibbs free energies were then calculated using the "direct method".[9] For systems that undergo large geometry changes upon solvation, the direct method affords energetics that are superior to those obtained using (gas-phase-to-solution) thermocycles.[9] Given the high partial charges on atoms within these metal complexes, significant geometry changes upon solvation (in water) would be anticipated. The default standard state used within ORCA 5.0.1. for entropic components (even in a CPCM solvent field) is based on the statistical mechanics for an ideal gas (evaluated at 1 atm of pressure and 25 °C). Thus, appropriate standard state corrections are applied to ensure all binding energies and pK_a values are calculated at a standard state for solutes in solution (of 1 mol L^{-1} and 25 °C). This correction takes the following form:

$$\Delta G^{1\mathrm{atm}\rightarrow 1\mathrm{M}} = \Delta m R T \ln\left[\frac{RT}{P}\right] \qquad \text{(Eq. 1)}$$

where Δm is the change in moles upon reaction, R is the ideal gas constant (8.3145 J mol^{-1} K^{-1}), T is the temperature of interest at which the Gibbs free energy is also evaluated (typically 298.15 K) and P is pressure (101.325 kPa = 1 atm).

For reactions that either generate or consume water (i.e., reactions where water is a product or reagent), a further state correction is required. The standard state for any liquid is the pure substance at 1 atm of pressure. Thus, the standard state of liquid water should be $[H_2O]$ = 55.5 mol L^{-1} (rather than 1 mol L^{-1} for an aqueous solute). Gibbs free energies for reactions involving water as a reagent must be further corrected by adding the following additional term:

$$\Delta G^{1\text{M}\rightarrow 55.5\text{M}} = -nRT\ln[\text{H}_2\text{O}] \qquad (\text{Eq. }2)$$

where *n* is the number of moles of water acting as a reagent. Conversely, for reactions that generate water as a product, Gibbs free energies are corrected by the following term:

$$\Delta G^{1\text{M}\rightarrow 55.5\text{M}} = +nRT\ln[\text{H}_2\text{O}] \qquad (\text{Eq. }3)$$

where *n* is the number of moles of water produced. We should emphasize that these standard state corrections are only required for reaction energies calculated via QM approaches. Molecular graphics were rendered with UCSF Chimera, developed by the Resource for Biocomputing, Visualization, and Informatics at the University of California, San Francisco, with support from NIH P41-GM103311.37.[10]

Calculation of pK_a Values and Binding Constants and Conformational Searching

The methodological approach used to determine pK_a values and binding constants was detailed in earlier work.[1] Briefly, pK_a values for Glu complexes were calculated using isodesmic proton-transfer approach using $[Fe^{II}(H_2O)_6]^{2+}$ and $[Fe^{II}(H_2O)_5(OH)]^+$ as references.[11] Corresponding experimental pK_a values for these Fe^{II} aqua species were taken from Ref [12]. Having determined the binding affinities for fully protonated Glu and the pK_a values for the respective Fe^{II}–Glu complexes, other energetic terms can be determined via Hess's law thermocycles. The major advantage of using a relative pK_a approach is that it corrects systematic errors in the metal–ligand deprotonation components caused by the continuum solvation model. These systematic errors would otherwise adversely affect the accuracy of binding parameters. For more details, regarding these considerations see discussion in Ref [1].

For all the Fe^{II} complexes derived from α- and β-Glu, a full-tree search was performed on the rotatable C–O and C–C bonds at a resolution of 120°. As illustrated in Figure S5, this affords the global minimum energy conformation at each binding position for the fully protonated species. As the stability of these fully protonated complexes is fairly insensitive to the conformational arrangement of the rotatable C–C and C–O bonds, analogous conformational searching for the deprotonated and doubly deprotonated complexes was not pursued.

As illustrated in Figure S5, binding at Sites 1–5 is fairly competitive (within 10 kJ mol^{-1}) for both α- and β-Glu. Thus, deprotonation of all these α- and β-Glu complexes was considered by sequentially removing a single proton from each of the Fe^{II}-bound O atoms and reoptimizing the resultant structure. This enables the most acidic proton for each complex to be identified, affording the deprotonated species depicted in Figure S6.

For a complex formed via chelation at Sites 1–4, the Fe^{II}-bound OH moieties of Glu are substantially more acidic than the aqua coligands (>15 kJ mol^{-1}). However, chelation at Site 5 positions a free OH moiety in close proximity to an Fe^{II}-bound aqua ligand, which enables the formation of a strong intramolecular H-bond upon aqua deprotonation. Deprotonation of the bound Glu OH group for these complexes was relatively less favorable (~10 kJ mol^{-1}) but is still considerably more favorable than ionization of non H-bonding aqua ligands (>20 kJ mol^{-1}). Given this distinct energetic preference for single deprotonation at the Fe^{II}-bound OH groups of Glu, as well as the proximate aqua ligand in the case of chelation at Site 5, only these positions were examined when considering double deprotonation.

As illustrated in Figure S7, the most favorable doubly deprotonated complex identified for α- and β-Glu involves Site 5 chelation. However, this protonation state was predicted to only be relevant at pH > 10.

QCM Analysis

Gold-coated QCM crystals were cleaned with Piranha solution (7:3 (v/v) 98% H_2SO_4/30% H_2O_2) before use. *Caution! Piranha solution is extremely corrosive and reacts violently with organic compounds! Extreme care should be taken during solution preparation and use!* The crystals were then extensively rinsed with Milli-Q water and dried under a stream of nitrogen. The precleaned gold-coated sensor crystals were mounted in sample chambers for each measurement, and the baseline was stabilized using pH 4 buffer (100 mM) for 10 min. The frequency values quoted were those of the third overtone. All sample injection and washing steps were performed at a speed of 200 μL min^{-1} by a peristaltic pump. After initially depositing a layer of MPN coating (mixed 10 mg mL^{-1} $FeCl_2\cdot 4H_2O$ and 5 mg mL^{-1} QUE in pH 4 buffer), different concentrations of Glu (5, 25, 50, or 100 mM) or 100 mM NaCl solution was sequentially injected and then the crystals were washed with pH 4 buffer until the frequency values stabilized. For the individual binding ability measurements, $FeCl_2\cdot 4H_2O$ (10 mg mL^{-1}) or QUE (5 mg mL^{-1}) was added to pH 4 buffer to keep the same molality, after which Glu solution was injected, followed by washing with pH 4 buffer until the frequency values stabilized.

Preparation of Fluorescein Isothiocyanate-Labeled Insulin (FINS)

On the basis of a previous report,[13] the preparation of FINS was followed. FITC solution in DMSO (5 mg mL^{-1}) was added dropwise with gentle stirring to human INS solution (2 mg mL^{-1}) in 0.01 M HCl solution (pH 3.5) at a molar ratio of 1:1. The reaction mixture was protected from light and kept at room temperature for 4 h with continuous slow stirring. Then, the reaction mixture was incubated at room temperature for 30 min without stirring. The solution was dialyzed against water to remove the unreacted FITC. The lyophilized FINS powder was stored at −20 °C for further use.

Preparation of Guest Molecule-Loaded MPN Capsules

The FINS-loaded MPN (FINS@MPN) capsules were prepared as follows: FINS (1 mg) was dissolved in 1 mM HCl (0.5 M NaCl, pH 3.5) to achieve a final concentration of 1 mg mL^{-1}. PS-COOH particles (30 μL) were washed twice with Milli-Q water (3000 *g*, 1 min) and suspended in water (250 μL). FINS solution (200 μL) was added to the suspension and stirred gently at room temperature for 30 min. Then, $FeCl_2\cdot 4H_2O$ (20 μL, 10 mg mL^{-1} in water) and QUE (30 μL, 5 mg mL^{-1} in methanol) were added successively to the mixture followed by brief vortexing and sonication. MOPS (500 μL,100 mM; pH 4) was added to adjust the pH of the mixed dispersion, followed by stirring at 650 rpm for 2 h to achieve sufficient film formation and adherence. The assembly of Fe^{II}−QUE MPN networks encapsulating

FINS was formed. For ζ-potential measurements, the INS-loaded particles were prepared by a similar route except that nonfluorescently labeled INS was added. To obtain the FINS@MPN capsules, the mixture was centrifuged (3000 *g*, 2 min) to remove noncoating complexes and the pellet was washed three times with Milli-Q water. Afterward, 1,4-dioxane (1000 μL) was added to dilute the suspension and for subsequent particle incubation for 1 h. Then, the pellets were washed four times with 1,4-dioxane (3000 *g*, 3 min), and the resulting capsules were resuspended in Milli-Q water for characterization. The encapsulation efficiency of FINS was determined by fluorescence spectrophotometry using a calibration curve of FINS.

$$\text{Encapsulation efficiency (\%)} = (\text{amount of encapsulated insulin}) / (\text{amount of insulin taken}) \times 100\% \qquad (\text{Eq. 4})$$

CYC-loaded MPN capsules were prepared by a similar route. The encapsulation efficiency of CYC was determined by UV−vis spectrophotometry using a calibration curve of CYC.

Stability of FINS@MPN Coatings in Different Solvents

The FINS@MPN particles and capsules were incubated in 100 mM urea, Tween 20, NaCl, DMSO, or DMF in an Eppendorf thermomixer at 37 °C and 650 rpm for 1.5 h. To assess the stability of the capsules, the suspensions were diluted with water and subjected to flow cytometry assays to determine the number of remaining capsules. The FINS@MPN particles in the above solvents were centrifuged (3000 *g*, 2 min) and washed three times with Milli-Q water, then redispersed in water. The fluorescence intensity of the particles was measured using a Nikon A1R+ laser scanning confocal microscope.

Glucose-Triggered Release of Guest Molecules from MPN Capsules

The FINS@MPN capsules were incubated in DPBS with Glu solution at different concentrations of 5, 25, and 50 mM and gently shaken at 37 °C. After incubation, the respective sample solutions were centrifuged at 3000 *g* for 5 min to precipitate the residual capsules, and the fluorescence of supernatants was measured on a fluorescence spectrophotometer at an excitation wavelength of 460 nm. The cumulative release was expressed as the total percentage of FINS released from capsules over time. To assess the self-regulated INS release profile, the FINS@MPN capsules were first incubated in Glu solution (1 mL, 400 mg dL^{-1}) for 30 min at 37 °C. The sample was then centrifuged at 3000 *g* for 5 min, and the supernatant was recovered. Next, the sample was incubated in Glu solution (1 mL, 100 mg dL^{-1}) for another 30 min. After centrifugation, the supernatant was recovered. This cycle was repeated thrice. The amount of the released INS was determined by spectrophotometry.

The release of CYC from the MPN capsules was triggered by 100 mM Glu, and the cumulative release profile was obtained by UV−vis spectrophotometry.

Visualization of FINS Release by STORM

The release of FINS from the MPN capsules was visualized on a Nikon N-STORM system equipped with a Nikon 100× 1.4 NA oil immersion objective. The focus and total internal reflection fluorescence imaging angle were adjusted to obtain a high signal-to-noise ratio. A 488 nm laser was used for the excitation of FINS. For STORM analysis,[14] FINS@MPN capsules were incubated in 50 mM Glu solution over time, and images were captured at 0, 4, and 8 h for number counting. The number of bound FINS molecules was quantified via intensity-based counting and an automatic count using the Nikon NIS elements software. All time lapses were recorded onto a 256 pixel × 256 pixel region using an electron-multiplying charge-coupled device camera. For each image, 3000−6000 frames were sequentially acquired, and images were analyzed using the N-STORM software ImageJ.

Conformation Analysis of INS

The secondary structure of the released INS was determined by the circular dichroism (CD) measurements. The standard INS was dissolved in DPBS solution to achieve a final concentration of 0.4 mg mL^{-1}. CD measurements were performed on a Jasco J-810 CD spectropolarimeter at 25 °C with a cell length of 0.1 cm. All samples were scanned from 190 to 260 nm and replicated three times, at a resolution of 1.0 nm and a scanning speed of 100 nm min^{-1}. All CD data were expressed as mean residue ellipticity.

Enzymatic and pH Stability of FINS@MPN Capsules

Pepsin, trypsin, and chymotrypsin were used as model proteases to investigate the enzymatic inhibition of FINS@MPN capsules in vitro. Pepsin was added to pH 2.0 buffers to obtain a simulated gastric fluid (SGF) solution, whereas trypsin and chymotrypsin were both added to pH 6.8 buffers to obtain simulated intestinal fluid (SIF) solutions. The capsules were sequentially added to SGF and subsequently to SIF solutions and DPBS (pH 7.4) buffers under constant stirring at 37 °C. At indicated time points, the number of capsules was counted using an Apogee A50-Micro flow cytometer (Apogee Flow Systems, UK). The data shown are the mean ± standard deviation from three independent measurements.

Cytotoxicity Assay of FINS@MPN Capsules

The (sodium 3′-[1-(phenylaminocarbonyl)-3,4-tetrazolium]-bis(4-methoxy-6-nitro) benzene sulfonic acid hydrate) (XTT)-based in vitro cytotoxicity assay was performed to assess the cell toxicity of FINS@MPN capsules. XTT was dissolved in complete DMEM (with 10% FBS) to obtain a 0.2 mg mL^{-1} solution, and phenazine methosulfate (PMS) was dissolved in DPBS to prepare a 1 mM solution. The XTT reagent was activated by mixing with PMS solution at a volume ratio of 400:1. 3T3 cells were seeded on a 96-well plate at a cell density of 2×10^4 cells per well. After incubation with FINS@MPN capsules at different capsule-to-cell ratios for 48 h, the media in the 96-well plate was aspirated and replaced with fresh activated XTT media (100 μL). The cells were further incubated for 4 h and the absorbance at 475 nm was measured relative to nontreated cells.

Cell Association of FINS@MPN Capsules

3T3 cells were seeded in a 24-well plate at a cell density of 10×10^4 cells per well and then cultured in complete media at 37 °C for 14 h to allow cellular adhesion on substrates. After incubation, FINS@MPN capsules were added to the cells at a capsule-to-cell ratio of 1000:1 and then incubated for 2, 4, 8, and 24 h at 37 °C. The percentage of cell association was calculated by measuring the fluorescence intensity of the cells. For CLSM imaging, 3T3 cells were seeded at 3×10^4 cells per well in Labtek 8-well chamber slides and incubated overnight. Then, FINS@MPN capsules were added to the cells at a capsule-to-cell ratio of 1000:1 and then incubated for 2, 4, 8, and 24 h at 37 °C. Following incubation, the cells were gently washed three times with DPBS, detached from the culture flask with trypsin, and collected by centrifugation (350 *g*, 5 min). Cells were then fixed with 4% paraformaldehyde (200 μL) for 15 min and gently washed three times with DPBS. The membrane was stained with Alex Fluor 549–wheat germ agglutinin (5 μg mL^{-1}) for 5

min, and the nucleus was stained with Hoechst 33342 (1 μg mL^{-1}) for 10 min. CLSM imaging was performed on a microscope with a 60 × 1.4 NA oil immersion objective. The images were processed by Fiji software.

In Vitro Transcytosis Studies

Caco-2 cells at a density of 30 × 10^4 cells per well were grown on 12-well transwell plates with 0.4 μm pore size (Costar 3460, Corning) for the development of a monolayer for 21 days. Before the experiment, Caco-2 monolayers were replaced with HBSS and allowed to equilibrate at 37 °C for 30 min. Afterward, the apical solution was replaced with fresh HBSS (200 μL) with the same molality of FINS@MPN capsules, free FINS, 70 kDa FITC−dextran, followed by incubation at 37 °C. FITC−dextran (70 kDa) was used to assess the integrity of the Caco-2 cell monolayer. At predetermined time intervals, the basolateral solution was collected, and the fluorescence intensity was measured by microplate at an excitation wavelength of 488 nm.

Minimum Information Reporting in Bio–Nano Experimental Literature (MIRIBEL)

The studies conducted herein, including material characterization, biological characterization, and experimental details, conform to the MIRIBEL reporting standard for bio–nano research,[15] and we include a companion checklist of these components herein.

Section S2. Supporting Figures

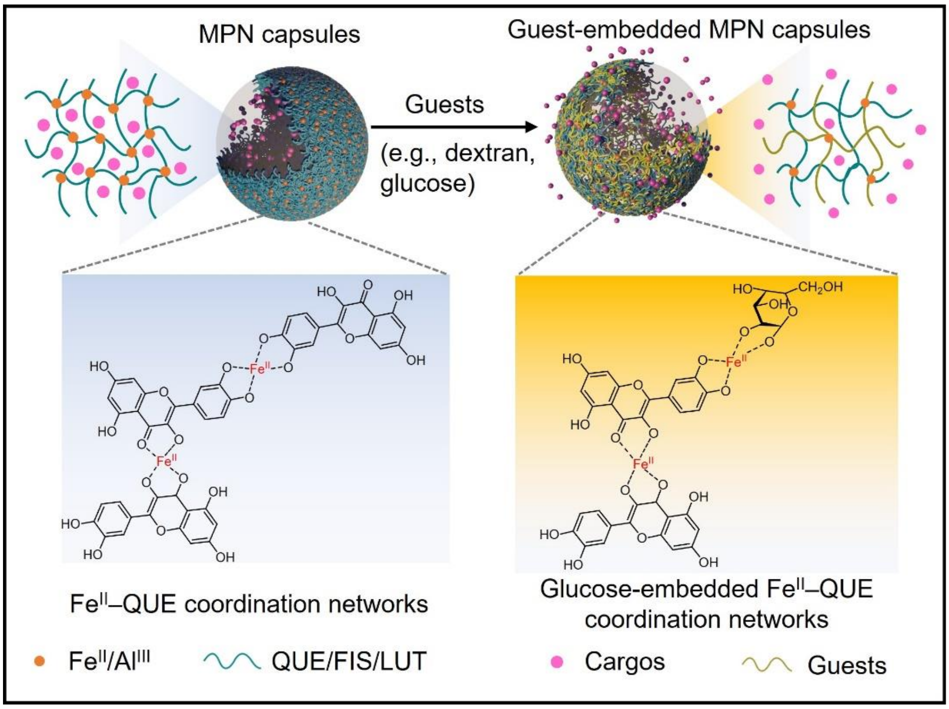

Figure S1. Schematic of the coordination networks of Fe^{II}–QUE MPN capsules and their structural reconfiguration via embedding guest molecules (e.g., glucose) when under external stimuli.

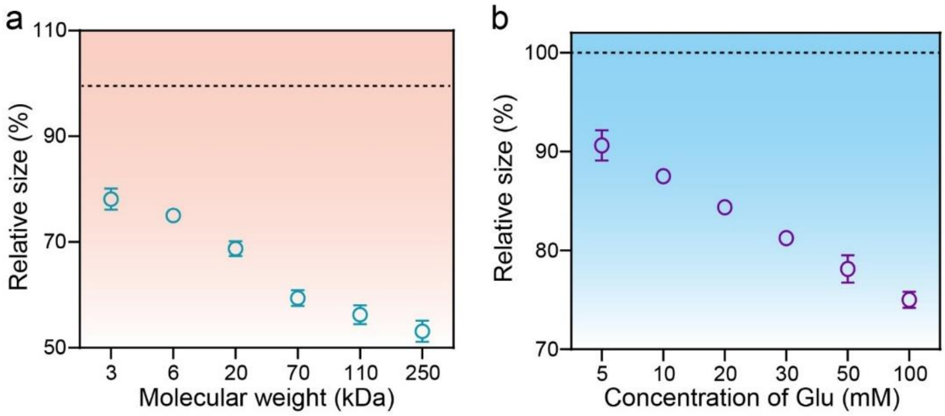

Figure S2. (a) Relative size of Fe^{II}–QUE capsules as a function of the molecular weight of dextran (3–250 kDa). (b) Relative size of Fe^{II}–QUE capsules when dispersed in Glu solutions at different concentrations. Twenty capsules of each sample were measured. The error bars represent standard deviations (n = 20). The dashed line represents the template size (i.e., 3.2 μm normalized to 100%), and the size (%) of the capsules is measured relative to the template size (100%).

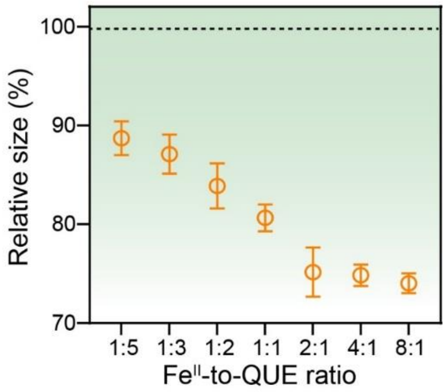

Figure S3. Relative size of capsules prepared under different Fe^{II}-to-QUE molar ratios upon incubation in 100 mM Glu. Twenty capsules of each sample were measured. The error bars represent standard deviations (*n* = 20). The dashed line represents the template size (i.e., 3.2 μm normalized to 100%), and the size (%) of the capsules is measured relative to the template size (100%).

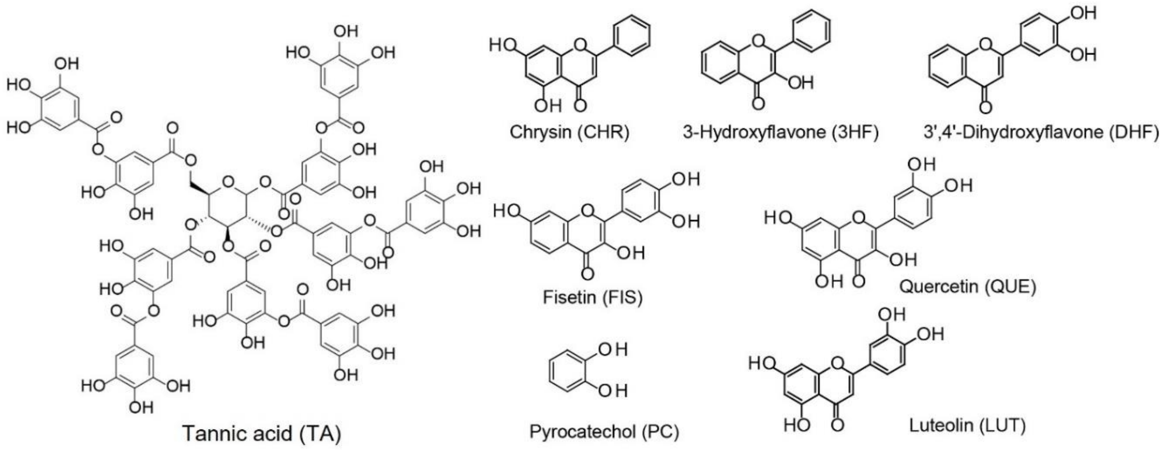

Figure S4. Chemical structures of phenolic ligands that were investigated in this study.

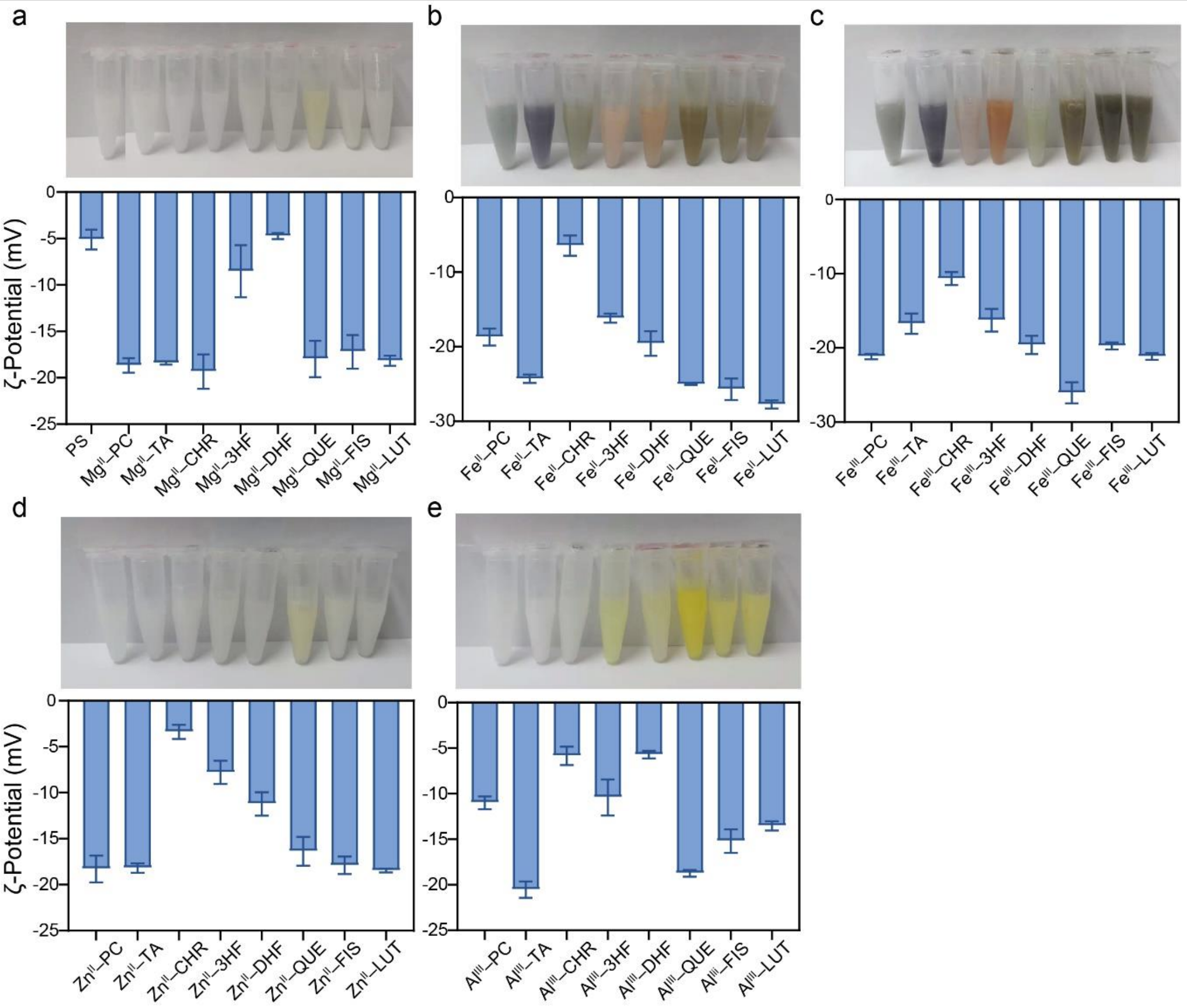

Figure S5. (a–e) Photographs and ζ-potential values of PS and PS@MPN coatings fabricated from a series of phenolic ligands and metal ions (Mg^{II} (a), Fe^{II} (b), Fe^{III} (c), Zn^{II} (d), and Al^{III} (e)). Data are shown as the mean ± standard deviation (n = 3).

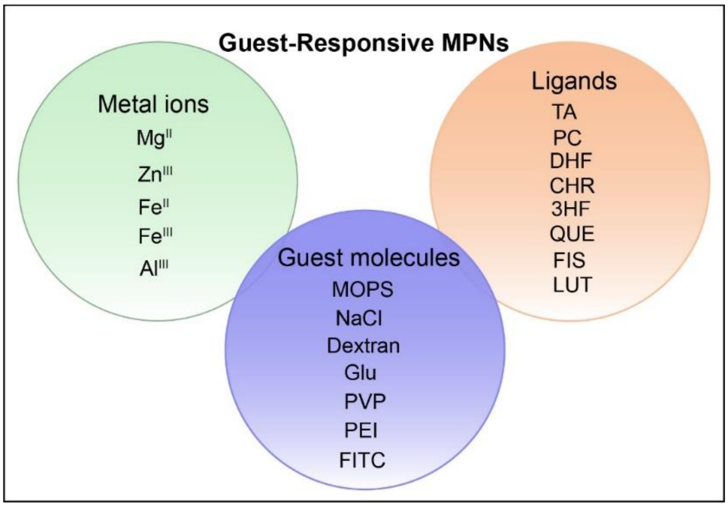

Figure S6. Schematic summarizing the various MPN building blocks (i.e., metal ions and phenolic ligands) and guest molecules that were used in this study.

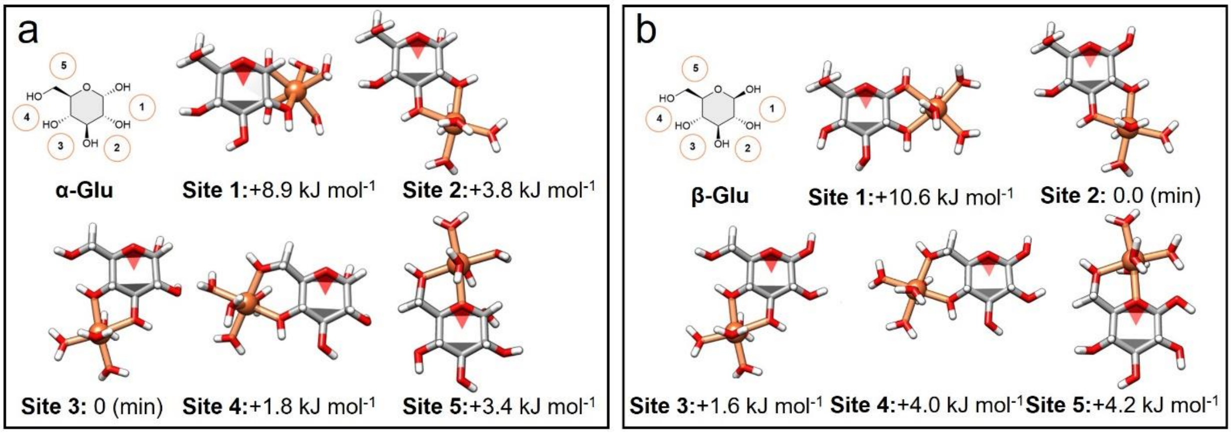

Figure S7. Relative Gibbs free energies for Fe^{II} complexation to the different sites of fully protonated α-Glu and β-Glu. All Gibbs free energies are normalized by minimal energy. Values are given in kJ mol^{-1} and calculated at 25 °C.

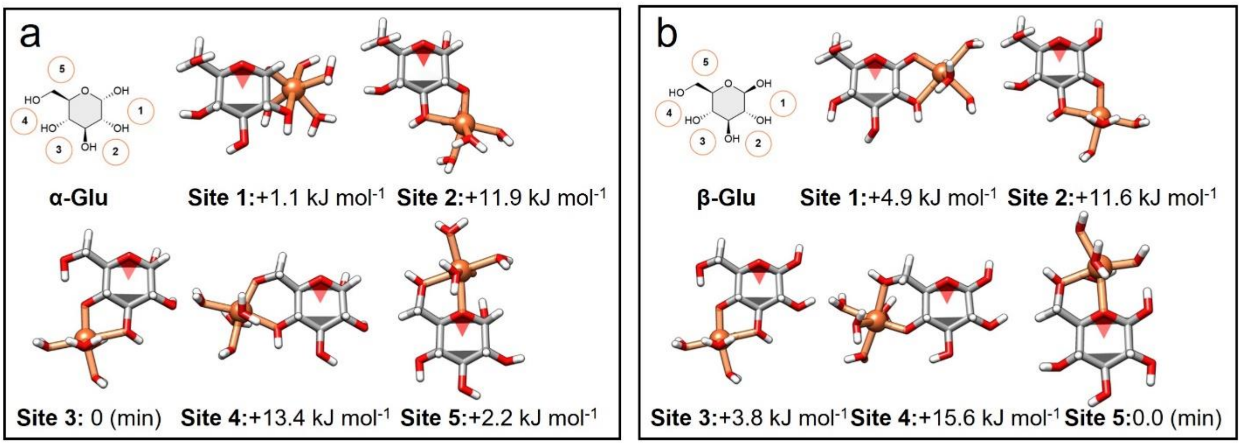

Figure S8. Relative Gibbs free energies for Fe^{II} complexation to the different sites of singly deprotonated α-Glu and β-Glu. Values are given in kJ mol^{-1} and calculated at 25 °C.

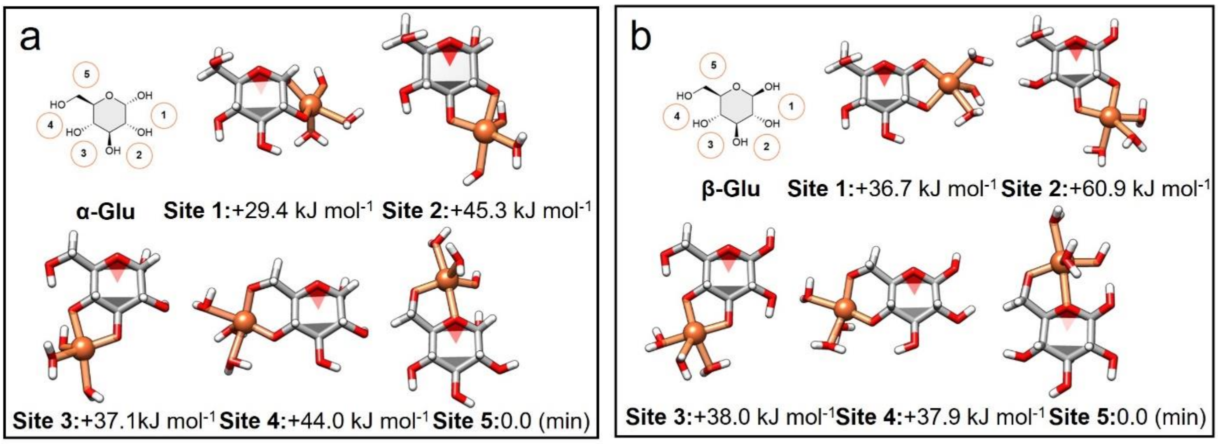

Figure S9. Relative Gibbs free energies for Fe^{II} complexation to the different sites of doubly deprotonated α-Glu and β-Glu. Values are given in kJ mol^{-1} and calculated at 25 °C.

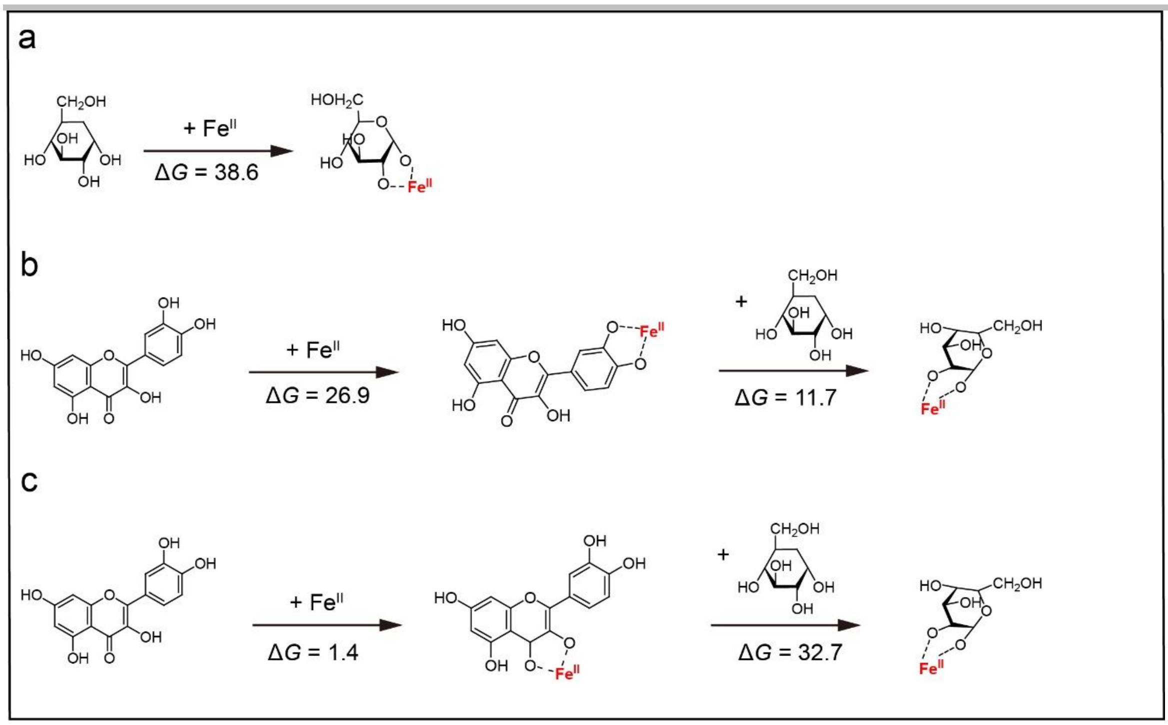

Figure S10. (a) Predicted Gibbs free energies for the direct chelation of Fe^{II} to Glu. (b,c) Predicted Gibbs free energies for the chelation of Fe^{II} to catechol site (b) or maltol site (c) of QUE and subsequent energy changes when QUE was partially replaced by Glu. The Gibbs free energies values (kJ mol^{-1}) were calculated at 25 °C. For clarity, water coligands and ligands displaced by exchange are not shown.

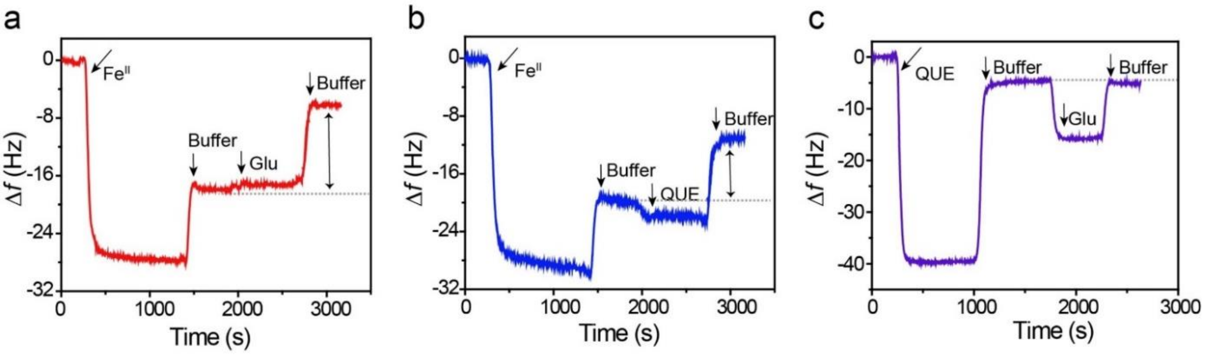

Figure S11. Binding kinetics of (a) Fe^{II} to Glu, (b) Fe^{II} to QUE, and (c) QUE to Glu, as monitored by QCM. The double arrow–dashed line indicates the Δ*f* obtained upon addition of Glu/QUE.

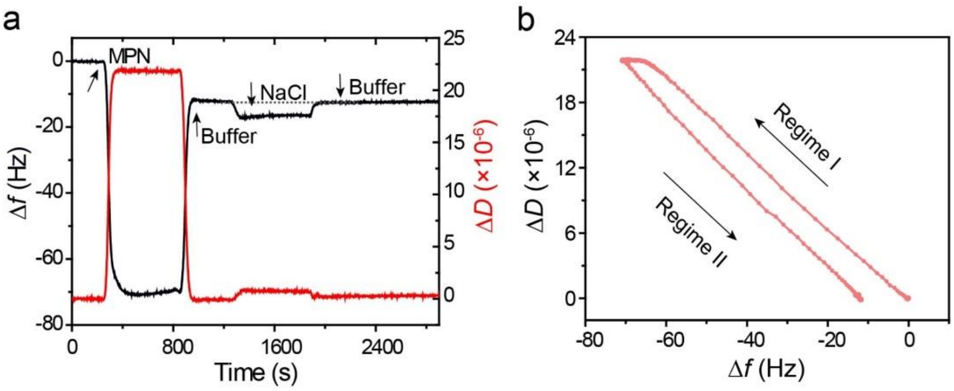

Figure S12. (a) Binding kinetics of MPNs to NaCl, as monitored by QCM. The double arrow–dashed line indicates the Δ*f* obtained upon addition of NaCl. (b) Adhesion dynamic between MPN and NaCl illustrated by Δ*D*–Δ*f* plot. Regime I represents the dynamic adsorption of NaCl onto MPN films and Regime II represents the detachment of NaCl from MPN films.

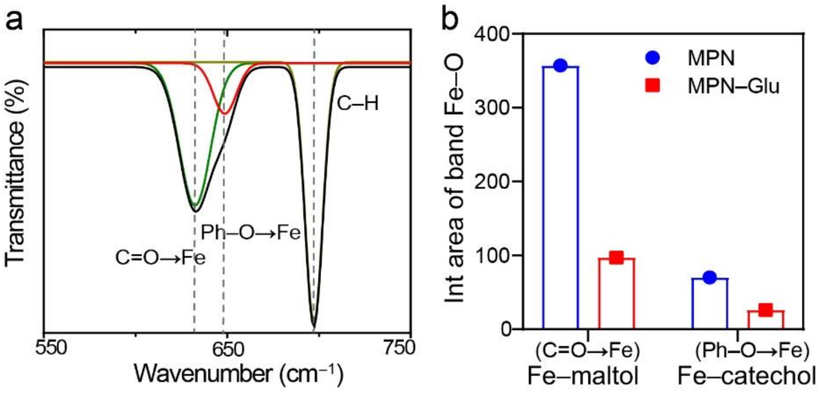

Figure S13. (a) Peak fitting of the FTIR spectra of the MPN–Glu capsules. The signals at 628, 645, and 700 nm were attributed to Fe–O bonds from carbonyl oxygen (C=O→Fe) or phenyl (Ph–O→Fe) and C–H stretching in the phenolic groups, respectively. (b) Integrated area of the fitted peaks of Fe–maltol (C=O→Fe) and Fe–catechol (Ph–O→Fe) groups within MPN and MPN–Glu capsules.

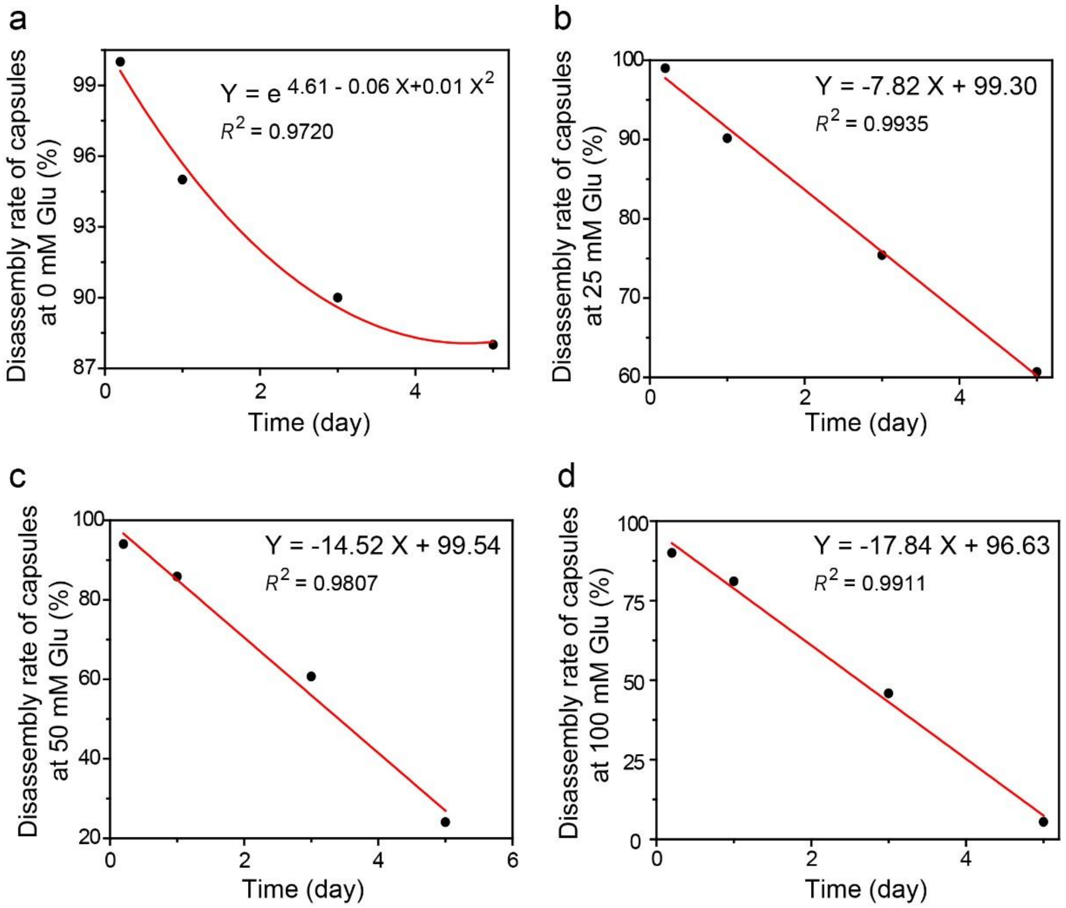

Figure S14. Disassembly kinetics of MPN capsules at different Glu concentrations: (a) 0 mM, (b) 25 mM, (c) 50 mM, and (d) 100 mM.

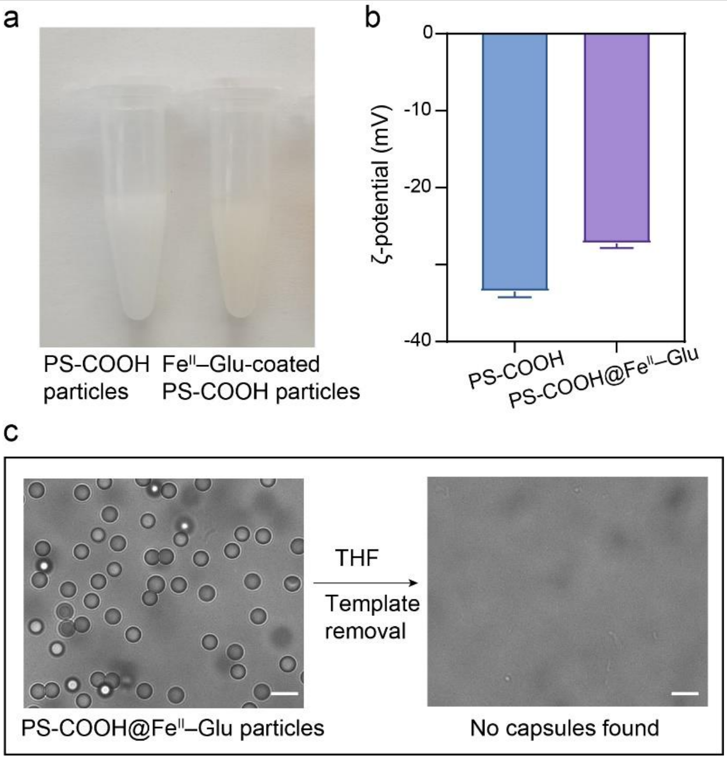

Figure S15. (a) Photograph and (b) ζ-potential values of PS-COOH and Fe^{II}–Glu-coated PS-COOH particles. (c) DIC images of Fe^{II}–Glu-coated PS-COOH particles before (left) and after (right) template removal. Scale bars are 3 μm.

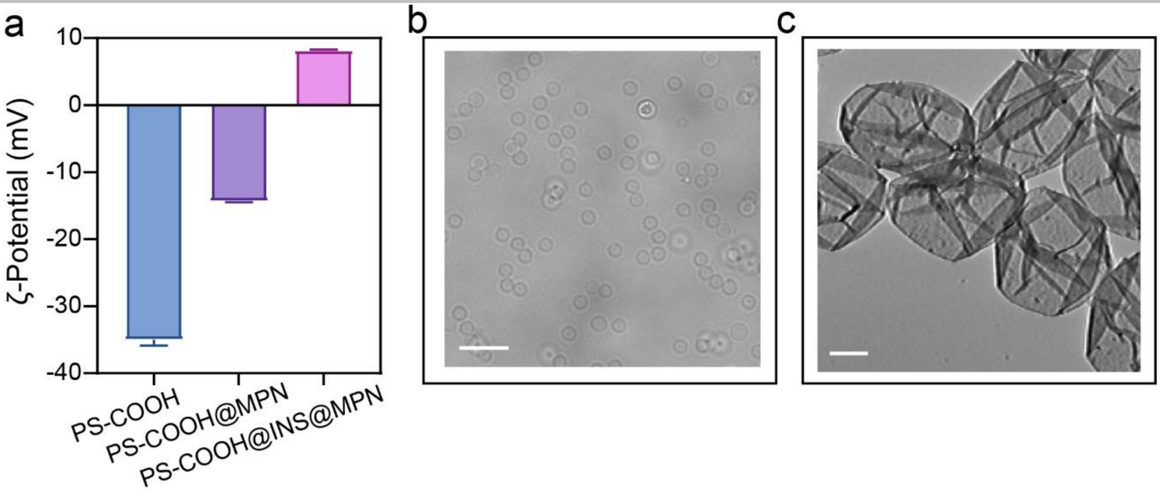

Figure S16. (a) ζ-Potential values of PS-COOH before and after coating measured in pH 4 buffer. DIC microscopy images showing (b) the well-dispersed INS@MPN capsules in water and (c) the air-dried capsules. Scale bars are 10 μm (b) and 1 μm (c).

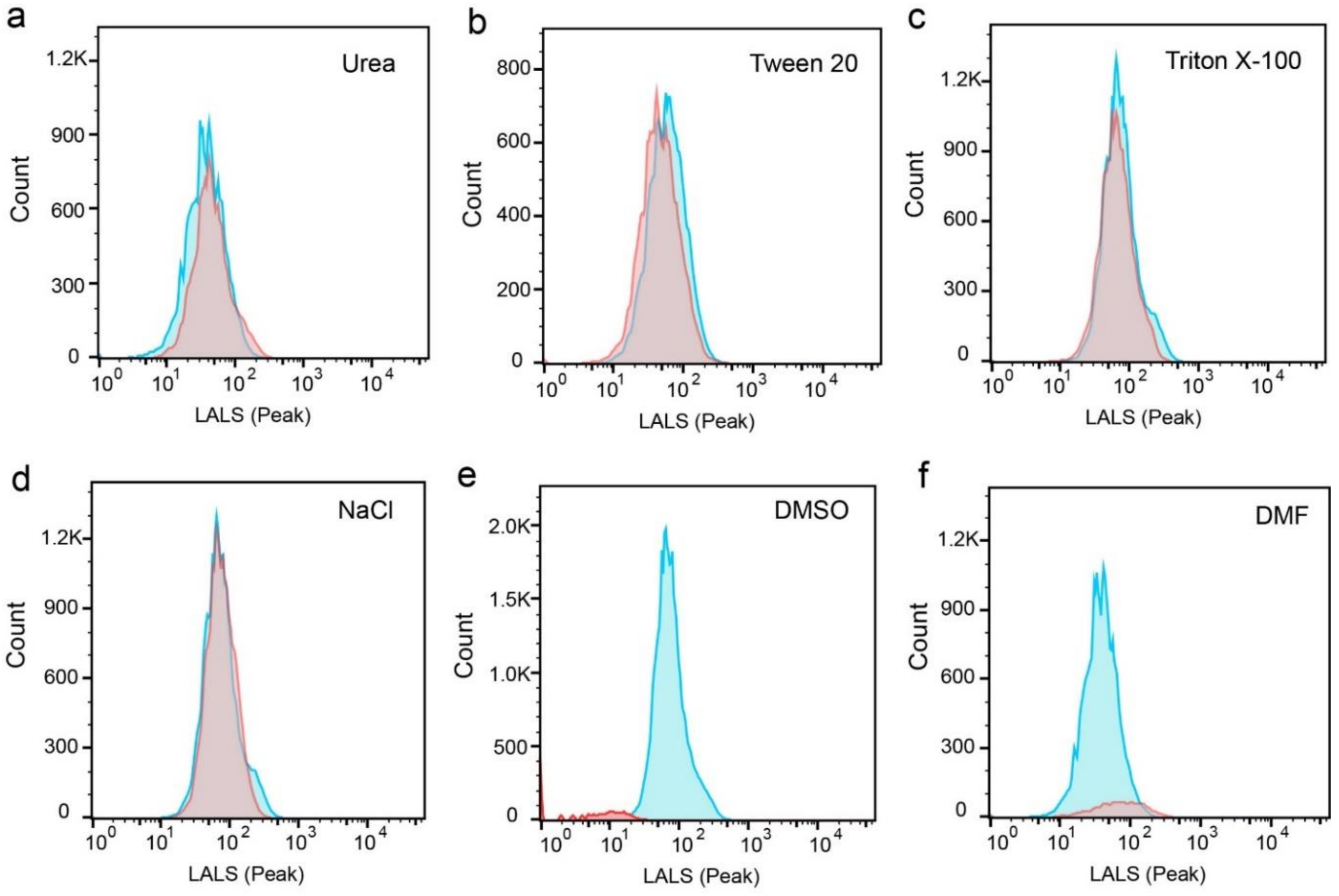

Figure S17. Histograms of the disassembly of FINS@MPN capsules in (a) 100 mM urea, (b) 100 mM Tween 20, (c) 100 mM Triton X-100, (d) 100 mM NaCl, (e) DMSO, or (f) DMF, as determined by flow cytometry. The blue curve was measured at 0 h and the red curve was measured at 1.5 h. LALS, low-angle light scattering (representing the capsule size).

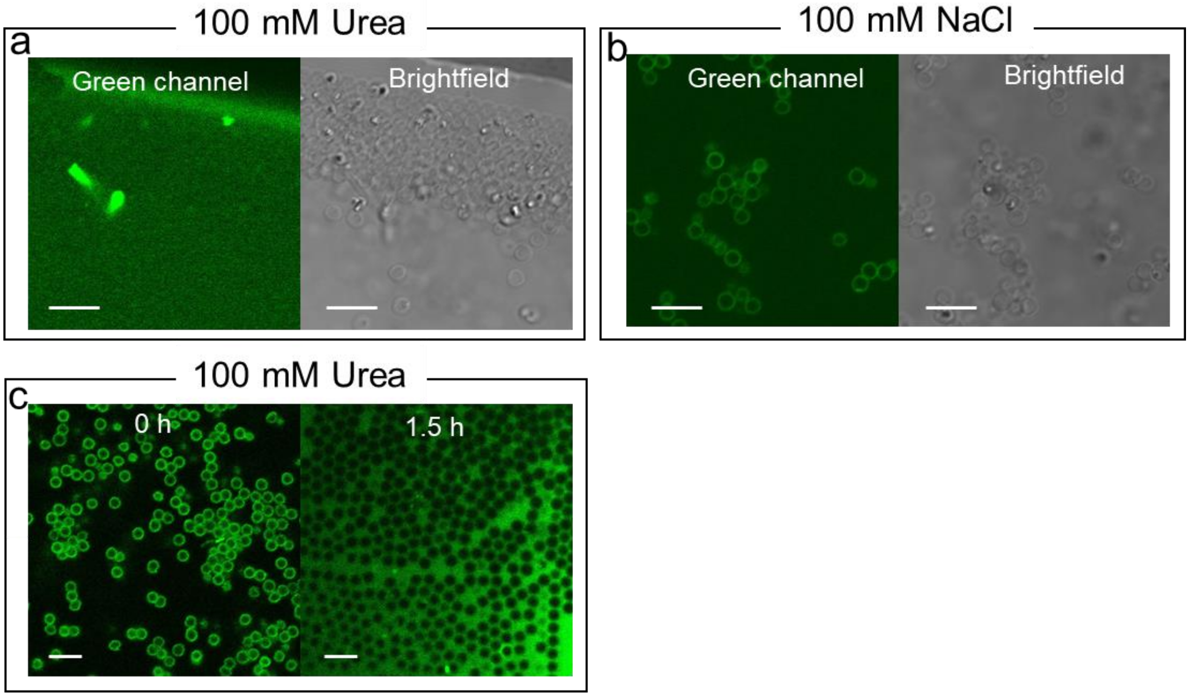

Figure S18. CLSM images of FINS@MPN capsules after incubation in (a) 100 mM urea or (b) 100 mM NaCl for 1.5 h. (c) CLSM images of PS-COOH@FINS@MPN particles after incubation in 100 mM urea for 0 and 1.5 h. Scale bars are 5 μm.

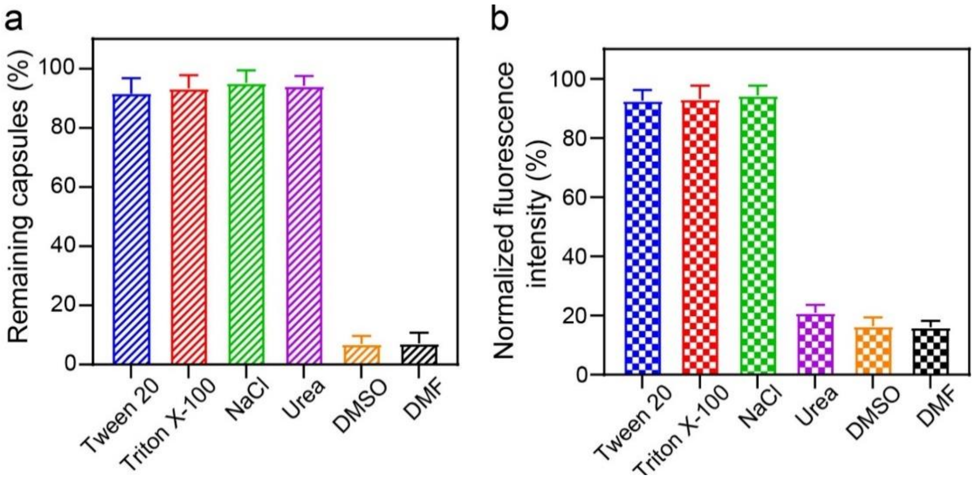

Figure S19. (a) Relative remaining quantity and (b) normalized fluorescence intensity of FINS@MPN capsules after incubation in 100 mM Tween 20, Triton X-100, NaCl, urea, DMSO, or DMF for 1.5 h. Data are shown as the mean ± standard deviation (n = 3).

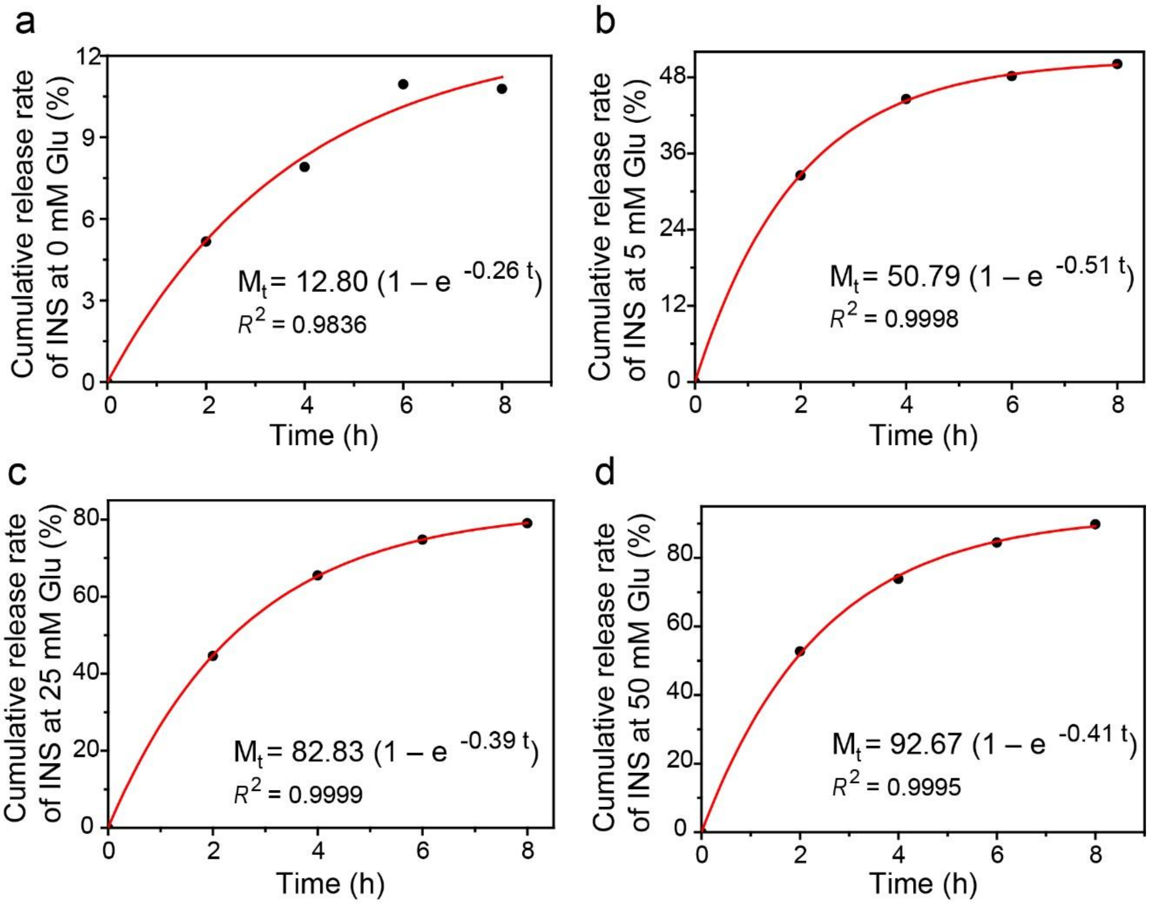

Figure S20. Release kinetics of INS from MPN capsules upon incubation with Glu at different concentrations: (a) 0 mM, (b) 5 mM, (c) 25 mM, and (d) 50 mM. M_t represents the cumulative release amount at time *t*.

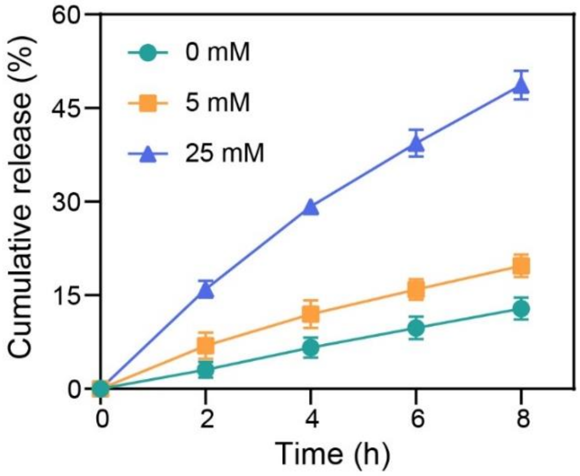

Figure S21. Cumulative release of INS from Al^{III}–QUE MPN capsules upon incubation with Glu solution at different concentrations over time. Data are shown as the mean ± standard deviation (*n* = 3).

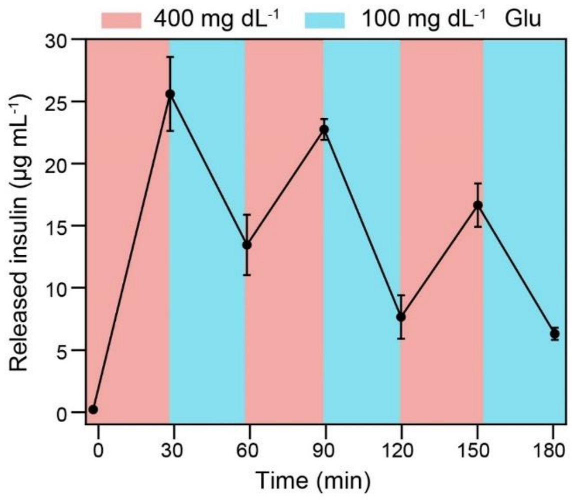

Figure S22. Pulsatile release of FINS from FINS@MPN capsules upon incubation with Glu solution at alternating concentrations of 100 and 400 mg dL^{-1}. Data are shown as the mean ± standard deviation (*n* = 3).

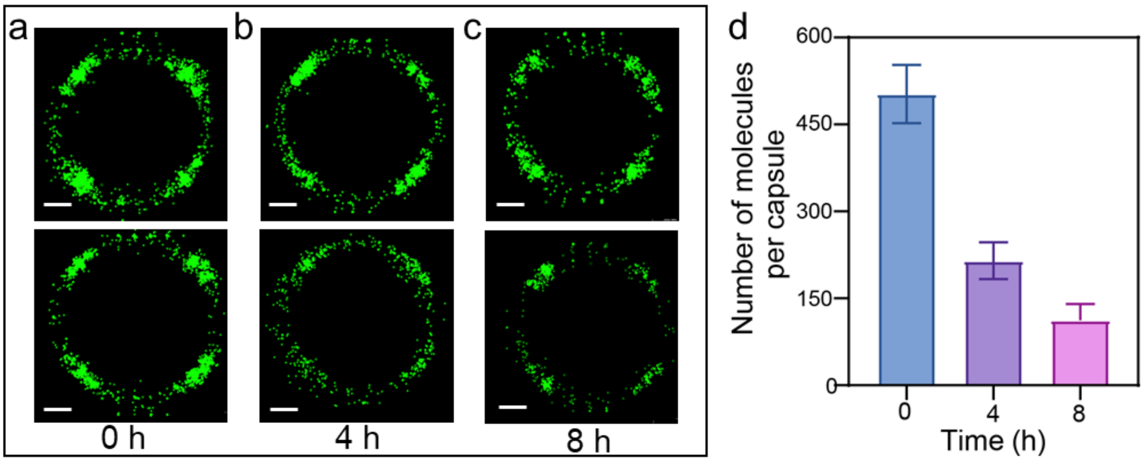

Figure S23. Super-resolution microscopy images of FINS@MPN capsules upon incubation in 50 mM Glu for different times: (a) 0, (b) 4, and (c) 8 h. (d) Number of FINS molecules identified in each FINS@MPN capsule after incubation in 50 mM Glu over time. Data are shown as the mean ± standard deviation (*n* = 50).

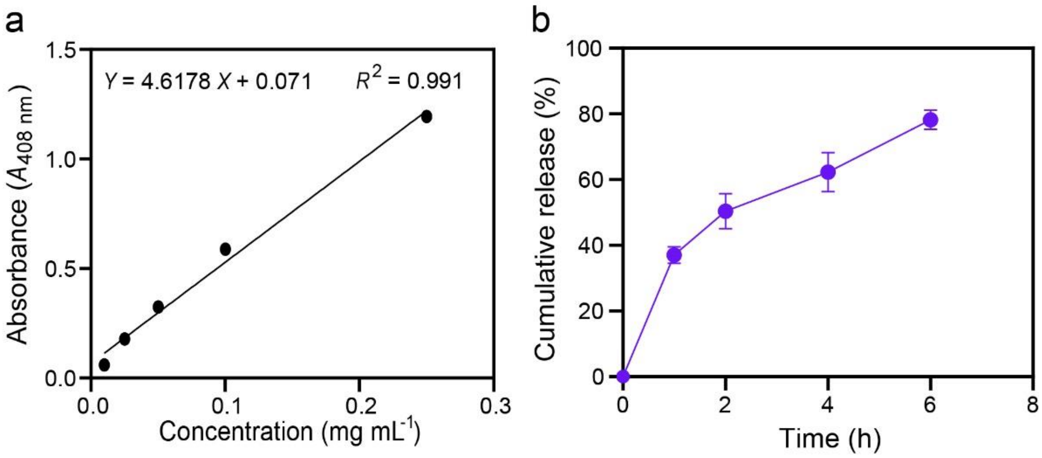

Figure S24. (a) UV–vis standard curve of CYC concentration at 408 nm. (b) Cumulative release of CYC from MPN capsules triggered by 100 mM Glu. Data are shown as the mean ± standard deviation (*n* = 3).

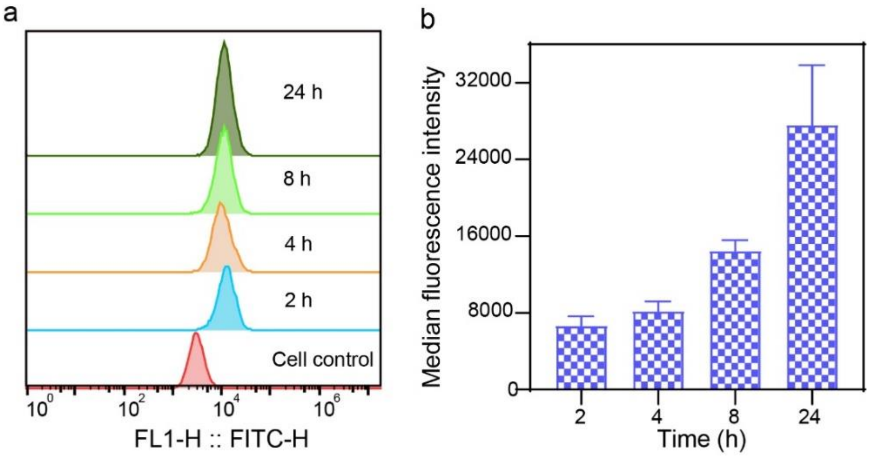

Figure S25. (a) Gating strategy and (b) mean fluorescence intensity of 3T3 cells treated with FINS@MPN capsules for 2, 4, 8, or 24 h at a capsule-to-cell ratio of 1000:1. Data are shown as the mean ± standard deviation (n = 3).

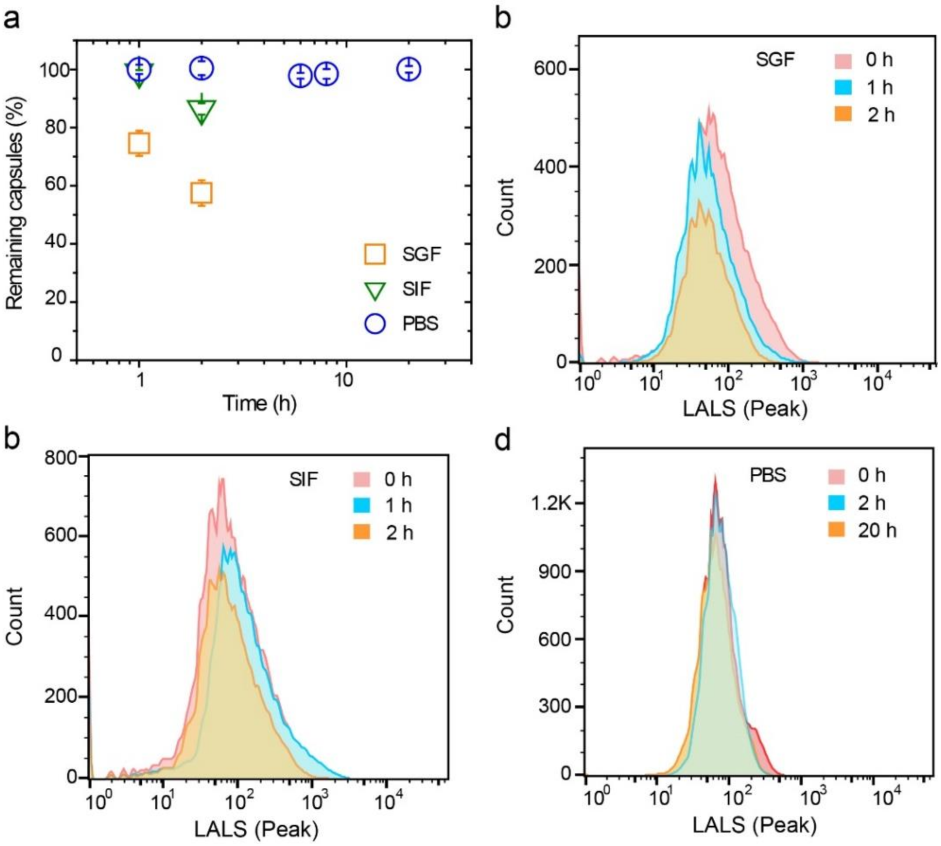

Figure S26. (a) Stability of FINS@MPN capsules in SGF, SIF, and PBS. Histograms of the stability of FINS@MPN capsules in (b) SGF, (c) SIF, and (d) PBS, as determined by flow cytometry. LALS, low-angle light scattering which represents the capsule size.

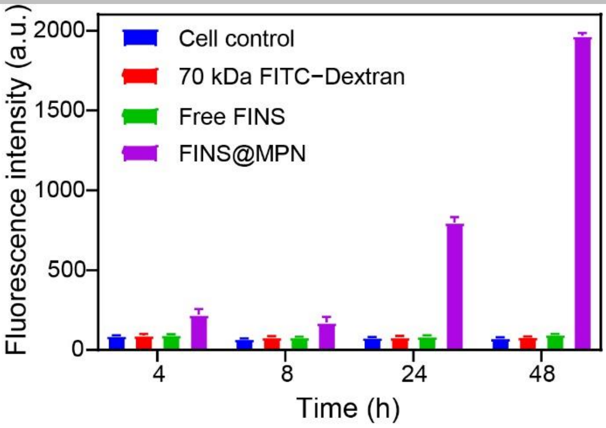

Figure S27. Cumulative transport of INS across Caco-2 cell monolayers. The fluorescence intensity was measured using a microplate reader at an excitation wavelength of 488 nm. FITC–dextran (70 kDa) was used to assess the integrity of the Caco-2 cell monolayer. Data are shown as the mean ± standard deviation (n = 3).

Section S3. Supporting Tables

Table S1. FTIR characteristic bands of MPN and MPN–Glu capsules[a]

Group assigned to the given band	Wavenumber (cm^{-1})	
	**MPN**	**MPN–Glu**
O–H stretching vibration of phenol	3293	3293
C=O aryl ketonic stretch	1640	1640
C=C aromatic ring stretching bands	1589, 1511, 1415	1591, 1513, 1413
O–H bending of phenol	1352	1354
C–H bonds in aromatic hydrocarbon bending (in-plane)	1312, 1116, 1094, 1015, 934	1310, 1073, 1015
C–O stretching of aryl ether (C–O–C)	1268	1267
C–O stretching in phenol	1198	1202
C–CO–C stretching and bending in ketones	1168	1165
C–H bending of aromatic hydrocarbons (out-of-plane)	808, 697	898, 807, 697
Fe–O stretching	625	625, 585

[a] Capsules were incubated with water and Glu solution and then freeze-dried before FTIR analysis.

Table S2. Integrated area of fitted peaks of MPN capsules in water and Glu[a]

**Capsules**	**Integrated area (C=O→Fe)**	**Integrated area (Ph–O→Fe)**	**Integrated area (C–C)**
MPN	357	70	100
MPN–Glu	97	26	100

[a] The Fe–O band can be attributed to binding to the carbonyl (C=O→Fe) and phenyl (Ph–O→Fe).

Table S3. Gibbs free energies for binding of $[Fe^{II}(H_2O)_6]^{2+}$ to α- and β-Glu and pK_a values of the resultant complexes[a]

Species	$\Delta G_{B/LH}$ (kJ mol^{-1})	ΔG_B* (kJ mol^{-1})	$\Delta G_{A/MLH}$ (kJ mol^{-1})	$-\log_{10}(K_B$*)	pK_a
$[Fe^{II}(\alpha\text{-}Glu_{s1})(H_2O)_4]^{2+}$	26.4	-	15.2	-	2.7
$[Fe^{II}(\alpha\text{-}Glu_{s1}^{-})(H_2O)_4]^{+}$	-	41.6	87.5	7.3	15.3
$[Fe^{II}(\alpha\text{-}Glu_{s1}^{2-})(H_2O)_3]^{0}$	-	129.2	-	22.6	-
$[Fe^{II}(\alpha\text{-}Glu_{s2})(H_2O)_4]^{2+}$	21.4	-	29.7	-	5.2
$[Fe^{II}(\alpha\text{-}Glu_{s2}^{-})(H_2O)_4]^{+}$	-	51.1	93.9	9.0	16.5
$[Fe^{II}(\alpha\text{-}Glu_{s2}^{2-})(H_2O)_3]^{0}$	-	145.1	-	25.4	-
$[Fe^{II}(\alpha\text{-}Glu_{s3})(H_2O)_4]^{2+}$	17.6	-	22.9	-	4.0
$[Fe^{II}(\alpha\text{-}Glu_{s3}^{-})(H_2O)_4]^{+}$	-	40.5	96.4	7.1	16.9
$[Fe^{II}(\alpha\text{-}Glu_{s3}^{2-})(H_2O)_3]^{0}$	-	136.9	-	24.0	-
$[Fe^{II}(\alpha\text{-}Glu_{s4})(H_2O)_4]^{2+}$	19.4	-	34.6	-	6.1
$[Fe^{II}(\alpha\text{-}Glu_{s4}^{-})(H_2O)_4]^{+}$	-	53.9	89.9	9.4	15.7
$[Fe^{II}(\alpha\text{-}Glu_{s4}^{2-})(H_2O)_3]^{0}$	-	143.8	-	25.2	-
$[Fe^{II}(\alpha\text{-}Glu_{s5})(H_2O)_4]^{2+}$	21.0	-	21.7	-	3.8
$[Fe^{II}(\alpha\text{-}Glu_{s5})(H_2O)_3(OH)]^{+}$	-	42.7	57.1	7.5	10.0
$[Fe^{II}(\alpha\text{-}Glu_{s5}^{-})(H_2O)_2(OH)]^{0}$	-	99.8	-	17.5	-
$[Fe^{II}(\beta\text{-}Glu_{s1})(H_2O)_4]^{2+}$	29.9	-	13.6	-	2.4
$[Fe^{II}(\beta\text{-}Glu_{s1}^{-})(H_2O)_4]^{+}$	-	43.5	89.9	7.6	15.7
$[Fe^{II}(\beta\text{-}Glu_{s1}^{2-})(H_2O)_3]^{0}$	-	133.4	-	23.4	-
$[Fe^{II}(\beta\text{-}Glu_{s2})(H_2O)_4]^{2+}$	19.4	-	30.8	-	5.4
$[Fe^{II}(\beta\text{-}Glu_{s2}^{-})(H_2O)_4]^{+}$	-	50.2	107.5	8.8	18.8
$[Fe^{II}(\beta\text{-}Glu_{s2}^{2-})(H_2O)_3]^{0}$	-	157.7	-	27.6	-
$[Fe^{II}(\beta\text{-}Glu_{s3})(H_2O)_4]^{2+}$	21.0	-	21.4	-	3.8
$[Fe^{II}(\beta\text{-}Glu_{s3}^{-})(H_2O)_4]^{+}$	-	42.4	92.3	7.4	16.2
$[Fe^{II}(\beta\text{-}Glu_{s3}^{2-})(H_2O)_3]^{0}$	-	134.7	-	23.6	-
$[Fe^{II}(\beta\text{-}Glu_{s4})(H_2O)_4]^{2+}$	23.4	-	30.8	-	5.4
$[Fe^{II}(\beta\text{-}Glu_{s4}^{-})(H_2O)_4]^{+}$	-	54.2	80.4	9.5	14.1
$[Fe^{II}(\beta\text{-}Glu_{s4}^{2-})(H_2O)_3]^{0}$	-	134.6	-	23.6	-
$[Fe^{II}(\beta\text{-}Glu_{s5})(H_2O)_4]^{2+}$	23.5	-	15.1	-	2.6
$[Fe^{II}(\beta\text{-}Glu_{s5})(H_2O)_3(OH)]^{+}$	-	38.6	58.1	6.8	10.2
$[Fe^{II}(\beta\text{-}Glu_{s5}^{-})(H_2O)_2(OH)]^{0}$	-	96.7	-	16.9	-

[a] Calculated at 25 °C. s1–s5 indicate the binding site. “-”, Not applicable.

Table S4. Raw energies of all the species in this study (all units are in Hartree)

Species	E_{sol} B97MV/def2-TZVP	G_{sol} B97MV/def2-TZVP	E_{sol} DLPNO-CCSD(T)/cc-pVTZ	G_{sol} Final Gibbs energy (Water)
H_2O	−76.443081	−76.439382	−76.341762	−76.338063
$[Fe^{II}(H_2O)_6]^{2+}$	−1721.93695	−1721.83328	−1720.94445	−1720.840783
$[Fe^{II}(H_2O)_5(OH)]^{+}$	−1721.47485	−1721.38395	−1720.47323	−1720.382328
$[Fe^{II}(H_2O)_3(OH)_2]^{0}$	−1644.55034	−1644.49418	−1643.64315	−1643.586983
α-Glu	−687.363322	−687.203202	−686.212393	−686.052273
β-Glu	−687.364961	−687.205095	−686.213092	−686.053226
$[Fe^{II}(\alpha\text{-Glu}_{s1})(H_2O)_4]^{2+}$	−2256.407330	−2256.160336	−2254.465417	−2254.218422
$[Fe^{II}(\alpha\text{-Glu}_{s2})(H_2O)_4]^{2+}$	−2256.408956	−2256.161578	−2254.467714	−2254.220337
$[Fe^{II}(\alpha\text{-Glu}_{s3})(H_2O)_4]^{2+}$	−2256.411444	−2256.162681	−2254.470556	−2254.221793
$[Fe^{II}(\alpha\text{-Glu}_{s4})(H_2O)_4]^{2+}$	−2256.408669	−2256.161965	−2254.467813	−2254.221108
$[Fe^{II}(\alpha\text{-Glu}_{s5})(H_2O)_4]^{2+}$	−2256.409887	−2256.162139	−2254.468243	−2254.220494
$[Fe^{II}(\alpha\text{-Glu}_{s1}^{-})(H_2O)_4]^{+}$	−2255.959749	−2255.725839	−2254.008759	−2253.774850
$[Fe^{II}(\alpha\text{-Glu}_{s2}^{-})(H_2O)_4]^{+}$	−2255.954534	−2255.721726	−2254.004039	−2253.771231
$[Fe^{II}(\alpha\text{-Glu}_{s3}^{-})(H_2O)_4]^{+}$	−2255.959072	−2255.724651	−2254.009705	−2253.775284
$[Fe^{II}(\alpha\text{-Glu}_{s4}^{-})(H_2O)_4]^{+}$	−2255.954900	−2255.721651	−2254.003413	−2253.770164
$[Fe^{II}(\alpha\text{-Glu}_{s5})(H_2O)_3(OH)]^{+}$	−2255.958694	−2255.724241	−2254.008905	−2253.774453
$[Fe^{II}(\alpha\text{-Glu}_{s1}^{2-})(H_2O)_3]^{0}$	−2179.028521	−2178.829497	−2177.169325	−2176.970301
$[Fe^{II}(\alpha\text{-Glu}_{s2}^{2-})(H_2O)_3]^{0}$	−2179.018938	−2178.820167	−2177.163006	−2176.964235
$[Fe^{II}(\alpha\text{-Glu}_{s3}^{2-})(H_2O)_3]^{0}$	−2179.022441	−2178.822624	−2177.167181	−2176.967364
$[Fe^{II}(\alpha\text{-Glu}_{s4}^{2-})(H_2O)_3]^{0}$	−2179.025108	−2178.824500	−2177.165322	−2176.964714
$[Fe^{II}(\alpha\text{-Glu}_{s5}^{-})(H_2O)_2(OH)]^{0}$	−2179.037730	−2178.838513	−2177.180697	−2176.981480
$[Fe^{II}(\beta\text{-Glu}_{s1})(H_2O)_4]^{2+}$	−2256.404325	−2256.158971	−2254.462446	−2254.217092
$[Fe^{II}(\beta\text{-Glu}_{s2})(H_2O)_4]^{2+}$	−2256.409654	−2256.163333	−2254.467434	−2254.221113
$[Fe^{II}(\beta\text{-Glu}_{s3})(H_2O)_4]^{2+}$	−2256.410396	−2256.162557	−2254.468329	−2254.220490
$[Fe^{II}(\beta\text{-Glu}_{s4})(H_2O)_4]^{2+}$	−2256.408552	−2256.161379	−2254.466752	−2254.219579
$[Fe^{II}(\beta\text{-Glu}_{s5})(H_2O)_4]^{2+}$	−2256.409205	−2256.161942	−2254.466792	−2254.219529
$[Fe^{II}(\beta\text{-Glu}_{s1}^{-})(H_2O)_4]^{+}$	−2255.956680	−2255.725068	−2254.005747	−2253.774135
$[Fe^{II}(\beta\text{-Glu}_{s2}^{-})(H_2O)_4]^{+}$	−2255.955647	−2255.723159	−2254.004071	−2253.771584
$[Fe^{II}(\beta\text{-Glu}_{s3}^{-})(H_2O)_4]^{+}$	−2255.958506	−2255.725091	−2254.007962	−2253.774547
$[Fe^{II}(\beta\text{-Glu}_{s4}^{-})(H_2O)_4]^{+}$	−2255.955222	−2255.721963	−2254.003316	−2253.770058
$[Fe^{II}(\beta\text{-Glu}_{s5})(H_2O)_3(OH)]^{+}$	−2255.959168	−2255.725616	−2254.009544	−2253.775992
$[Fe^{II}(\beta\text{-Glu}_{s1}^{2-})(H_2O)_3]^{0}$	−2179.025803	−2178.827876	−2177.166610	−2176.968683
$[Fe^{II}(\beta\text{-Glu}_{s2}^{2-})(H_2O)_3]^{0}$	−2179.018903	−2178.819238	−2177.159104	−2176.959439
$[Fe^{II}(\beta\text{-Glu}_{s3}^{2-})(H_2O)_3]^{0}$	−2179.025553	−2178.826610	−2177.167133	−2176.968190
$[Fe^{II}(\beta\text{-Glu}_{s4}^{2-})(H_2O)_3]^{0}$	−2179.027985	−2178.829451	−2177.166752	−2176.968218
$[Fe^{II}(\beta\text{-Glu}_{s5}^{-})(H_2O)_2(OH)]^{0}$	−2179.039224	−2178.840443	−2177.181431	−2176.982650

Section S4. Additional Information

Checklist

Minimum Information Reporting in Bio–Nano Experimental Literature

The MIRIBEL guidelines were introduced here: https://doi.org/10.1038/s41565-018-0246-4

The development of these guidelines was led by the ARC Centre of Excellence in Convergent Bio-Nano Science and Technology: https://www.cbns.org.au/. Any updates or revisions to this document will be made available here: http://doi.org/10.17605/OSF.IO/SMVTF. This document is made available under a CC-BY 4.0 license: https://creativecommons.org/licenses/by/4.0/.

The MIRIBEL guidelines were developed to facilitate reporting and dissemination of research in bio–nano science. Their development was inspired by various similar efforts:

- MIAME (microarray experiments): *Nat. Genet.* **29** (2001), 365; http://doi.org/10.1038/ng1201-365
- MIRIAM (biochemical models): *Nat. Biotechnol.* **23** (2005) 1509; http://doi.org/10.1038/nbt1156
- MIBBI (biology/biomedicine): *Nat. Biotechnol.* **26** (2008) 889; http://doi.org/10.1038/nbt.1411
- MIGS (genome sequencing): *Nat. Biotechnol.* **26** (2008) 541; http://doi.org/10.1038/nbt1360
- MIQE (quantitative PCR): *Clin. Chem.* **55** (2009) 611; http://doi.org/10.1373/clinchem.2008.112797
- ARRIVE (animal research): *PLOS Biol.* **8** (2010) e1000412; http://doi.org/10.1371/journal.pbio.1000412
- *Nature*'s reporting standards:
    - Life science: https://www.nature.com/authors/policies/reporting.pdf; e.g., *Nat. Nanotechnol.* **9** (2014) 949; http://doi.org/10.1038/nnano.2014.287
    - Solar cells: https://www.nature.com/authors/policies/solarchecklist.pdf; e.g., *Nat. Photonics* **9** (2015) 703; http://doi.org/10.1038/nphoton.2015.233
    - Lasers: https://www.nature.com/authors/policies/laserchecklist.pdf; e.g., *Nat. Photonics* **11** (2017) 139; http://doi.org/10.1038/nphoton.2017.28
- The "TOP guidelines": e.g., *Science* **352** (2016) 1147; http://doi.org/10.1126/science.aag2359

Similar to many of the efforts listed above, the parameters included in this checklist are **not** intended to be definitive requirements; instead they are intended as 'points to be considered', with authors themselves deciding which parameters are—and which are not—appropriate for their specific study.

This document is intended to be a living document, which we propose is revisited and amended annually by interested members of the community, who are encouraged to contact the authors of this document. Parts of this document were developed at the annual International Nanomedicine Conference in Sydney, Australia: http://www.oznanomed.org/, which will continue to act as a venue for their review and development, and interested members of the community are encouraged to attend.

After filling out the following pages, this checklist document can be attached as a "Supporting Information" document during submission of a manuscript to inform Editors and Reviewers (and eventually readers) that all points of MIRIBEL have been considered.

Supplementary Table 1. Material characterization*

**Question**	**Yes**	**No**
1.1 Are “**best reporting practices**” **available** for the nanomaterial used? For examples, see *Chem. Mater.* **28** (2016) 3535; http://doi.org/10.1021/acs.chemmater.6b01854 and *Chem. Mater.* **29** (2017) 1; http://doi.org/10.1021/acs.chemmater.6b05235	Not applicable	
1.2 If they are available, **are they used**? If not available, ignore this question and proceed to the next one.		
1.3 Are extensive and clear instructions reported detailing all steps of **synthesis** and the resulting **composition** of the nanomaterial? For examples, see *Chem. Mater.* **26** (2014) 1765; http://doi.org/10.1021/cm500632c, and *Chem. Mater.* **26** (2014) 2211; http://doi.org/10.1021/cm5010449. Extensive use of photos, images, and videos are strongly encouraged. For example, see *Chem. Mater.* **28** (2016) 8441; http://doi.org/10.1021/acs.chemmater.6b04639	√	
1.4 Is the **size** (or **dimensions**, if non-spherical) and **shape of** the nanomaterial reported?	√	
1.5 Is the **size dispersity** or **aggregation** of the nanomaterial reported?	√	
1.6 Is the **zeta potential** of the nanomaterial reported?	√	
1.7 Is the **density (mass/volume)** of the nanomaterial reported?	√	
1.8 Is the amount of any **drug loaded** reported? ‘Drug’ here broadly refers to functional cargos (e.g., proteins, small molecules, nucleic acids).	√	
1.9 Is the **targeting performance** of the nanomaterial reported, including **amount** of ligand bound to the nanomaterial if the material has been functionalised through addition of targeting ligands?	Not applicable	
1.10 Is the **label signal** per nanomaterial/particle reported? For example, fluorescence signal per particle for fluorescently labelled nanomaterials.	√	
1.11 If a material property not listed here is varied, has it been **quantified**?	√	
1.12 Were characterizations performed in a **fluid mimicking biological conditions**?	Not applicable	
1.13 Are details of how these parameters were **measured/estimated** provided?	√	
Explanation for **No** (if needed):		

***Ideally, material characterization should be performed in the same biological environment as that in which the study will be conducted. For example, for cell culture studies with nanoparticles, characterization steps would ideally be performed on nanoparticles dispersed in cell culture media. If this is not possible, then characteristics of the dispersant used (e.g., pH, ionic strength) should mimic as much as possible the biological environment being studied.

Supplementary Table 2. Biological characterization*

**Question**	**Yes**	**No**
2.1 Are **cell seeding details**, including **number of cells plated, confluency at start of experiment,** and **time between seeding and experiment** reported?	√	
2.2 If a standardised cell line is used, are the **designation and source** provided?	√	
2.3 Is the **passage number** (total number of times a cell culture has been subcultured) known and reported?	√	
2.4 Is the last instance of **verification of cell line** reported? If no verification has been performed, is the time passed and passage number since acquisition from trusted source (e.g., ATCC or ECACC) reported? For information, see *Science* **347** (2015) 938; http://doi.org/10.1126/science.347.6225.938	√	
2.5 Are the results from **mycoplasma testing** of cell cultures reported?	√	
2.6 Is the **background signal of cells/tissue** reported? (E.g., the fluorescence signal of cells without particles in the case of a flow cytometry experiment.)	√	
2.7 Are **toxicity studies** provided to demonstrate that the material has the expected toxicity, and that the experimental protocol followed does not?	√	
2.8 Are details of media preparation (**type of media, serum,** any **added antibiotics**) provided?	√	
2.9 Is a **justification of the biological model** used provided? For examples for cancer models, see *Cancer Res.* **75** (2015) 4016; http://doi.org/10.1158/0008-5472.CAN-15-1558, and *Mol. Ther.* **20** (2012) 882; http://doi.org/10.1038/mt.2012.73, and *ACS Nano* **11** (2017) 9594; http://doi.org/10.1021/acsnano.7b04855	√	
2.10 Is characterization of the **biological fluid** (*ex vivo*/*in vitro*) reported? For example, when investigating protein adsorption onto nanoparticles dispersed in blood serum, pertinent aspects of the blood serum should be characterised (e.g., protein concentrations and differences between donors used in study).	Not applicable	
2.11 For **animal experiments**, are the ARRIVE guidelines followed? For details, see *PLOS Biol.* **8** (2010) e1000412; http://doi.org/10.1371/journal.pbio.1000412	Not applicable	
Explanation for **No** (if needed):		

*For *in vitro* experiments (e.g., cell culture), *ex vivo* experiments (e.g., in blood samples), and *in vivo* experiments (e.g., animal models). The questions above that are appropriate depend on the type of experiment conducted.

Supplementary Table 3. Experimental details*

**Question**	**Yes**	**No**
3.1 For cell culture experiments: are **cell culture dimensions** including **type of well**, **volume of added media**, reported? Are cell types (i.e.; adherent vs suspension) and **orientation** (if non-standard) reported?	√	
3.2 Is the **dose of material administered** reported? This is typically provided in nanomaterial mass, volume, number, or surface area added. Is sufficient information reported so that regardless of which one is provided, the other dosage metrics can be calculated (i.e. using the dimensions and density of the nanomaterial)?	√	
3.3 For each type of imaging performed, are details of how **imaging** was performed provided, including details of **shielding, non-uniform image processing**, and any **contrast agents** added?	√	
3.4 Are details of how the dose was administered provided, including **method of administration, injection location, rate of administration,** and details of **multiple injections**?	√	
3.5 Is the methodology used to **equalise dosage** provided?	√	
3.6 Is the **delivered dose** to tissues and/or organs (in vivo) reported, as % injected dose per gram of tissue (%ID g^{-1})?	Not applicable	
3.7 Is **mass of each organ/tissue measured** and **mass of material** reported?	Not applicable	
3.8 Are the **signals of cells/tissues with nanomaterials** reported? For instance, for fluorescently labelled nanoparticles, the total number of particles per cell or the fluorescence intensity of particles + cells, at each assessed timepoint.	√	
3.9 Are **data analysis details**, including **code used** for analysis provided?	√	
3.10 Is the **raw data** or **distribution of values** underlying the reported results provided? For examples, see *R. Soc. Open Sci.* **3** (2016) 150547; http://doi.org/10.1098/rsos.150547, https://opennessinitiative.org/making-your-data-public/, http://journals.plos.org/plosone/s/data-availability, and https://www.nature.com/sdata/policies/repositories	√	
Explanation for **No** (if needed):		

* The use of protocol repositories (e.g., *Protocol Exchange* http://www.nature.com/protocolexchange/) and published standard methods and protocols (e.g., *Chem. Mater.* **29** (2017) 1; http://doi.org/10.1021/acs.chemmater.6b05235, and *Chem. Mater.* **29** (2017) 475; http://doi.org/10.1021/acs.chemmater.6b05481) are encouraged.

Section S5. Supporting References

[1] W. Xu, S. Pan, B. B. Noble, J. Chen, Z. Lin, Y. Han, J. Zhou, J. J. Richardson, I. Yarovsky, F. Caruso, *Angew. Chem. Int. Ed.* **2022**, *61*, e202208037; *Angew. Chem.* **2022**, *134*, e202208037.
[2] F. Neese, *WIREs Comput. Mol. Sci.* **2012**, *2*, 73–78.
[3] N. Mardirossian, M. Head-Gordon, *J. Chem. Phys.* **2015**, *142*, 074111.
[4] F. Weigend, R. Ahlrichs, *Phys. Chem. Chem. Phys.* **2005**, *7*, 3297–3305.
[5] F. Weigend, *Phys. Chem. Chem. Phys.* **2006**, *8*, 1057–1065.
[6] V. Barone, M. Cossi, *J. Phys. Chem. A* **1998**, *102*, 1995–2001.
[7] a) T. H. Dunning Jr., *J. Chem. Phys.* **1989**, *90*, 1007–1023; b) N. B. Balabanov, K. A. Peterson, *J. Chem. Phys.* **2005**, *123*, 064107.
[8] a) F. Weigend, A. Köhn, C. Hättig, *J. Chem. Phys.* **2002**, *116*, 3175–3138; b) J. G. Hill, J. A. Platts, *J. Chem. Phys.* **2008**, *129*, 134101.
[9] J. Ho, *Phys. Chem. Chem. Phys.* **2015**, *17*, 2859–2868.
[10] E. F. Pettersen, T. D. Goddard, C. C. Huang, G. S. Couch, D. M. Greenblatt, E. C. Meng, T. E. Ferrin, *J. Comput. Chem.* **2004**, *25*, 1605–1612.
[11] J. Ho, M. L. Coote, *Theor. Chem. Acc.* **2009**, *125*, 3.
[12] a) F. J. Millero, W. Yao, J. Aicher, *Mar. Chem.* **1995**, *50*, 21–39; b) F. J. Millero, D. J. Hawke, *Mar. Chem.* **1992**, *40*, 19–48.
[13] D. Shah, Y. Guo, J. Ocando, J. Shao, *J. Pharm. Anal.* **2019**, *9*, 400–405.
[14] A. Radziwon, S. K. Bhangu, S. Fernandes, C. Cortez-Jugo, R. De Rose, B. Dyett, M. Wojnilowicz, P. Laznickova, J. Fric, G. J. N. Forte, **2022**, *14*, 3452–3466.
[15] M. Faria, M. Björnmalm, K. J. Thurecht, S. J. Kent, R. G. Parton, M. Kavallaris, A. P. R. Johnston, J. J. Gooding, S. R. Corrie, B. J. Boyd, P. Thordarson, A. K. Whittaker, M. M. Stevens, C. A. Prestidge, C. J. H. Porter, W. J. Parak, T. P. Davis, E. J. Crampin, F. Caruso, *Nat. Nanotechnol.* **2018**, *13*, 777–785.

Author Contributions

W.X., S.P., and F.C. conceived the ideas, designed, and led the project. All authors performed research and/or analyzed data with intellectual contributions. W.X., S.P., Z.L., and F.C. drafted the manuscript with intellectual input from all authors. The authors declare no conflict of interest.
